# Prediction of Novel Drug Targets and Vaccine Candidates against Human Lice (Insecta), Acari (Arachnida), and Their Associated Pathogens

**DOI:** 10.3390/vaccines10010008

**Published:** 2021-12-22

**Authors:** Abid Ali, Shabir Ahmad, Pedro Machado Medeiros de Albuquerque, Atif Kamil, Fahdah Ayed Alshammari, Abdulaziz Alouffi, Itabajara da Silva Vaz

**Affiliations:** 1 Department of Zoology, Abdul Wali Khan University Mardan, Mardan 23200, Pakistan; drabid@awkum.edu.pk; 2 Centro de Biotecnologia, Universidade Federal do Rio Grande do Sul, Porto Alegre 91501-970, Brazil; shabir.ahmad@ufrgs.br (S.A.); pedro.machado@ufrgs.br (P.M.M.d.A.); 3 Department of Biotechnology, Abdul Wali Khan University Mardan, Mardan 23200, Pakistan; atifkamil@awkum.edu.pk; 4 College of Sciences and Literature Microbiology, Nothern Border University, Rafha 76413, Saudi Arabia; fahdah.ayed@nbu.edu.sa; 5 King Abdulaziz City for Science and Technology, Riyadh 12354, Saudi Arabia; aaloufi@kacst.edu.sa; 6 Vaccines Research for Infectious Diseases, King Saud University, Riyadh 11495, Saudi Arabia; 7 Veterinary Laboratories and Vaccines Center, Ministry of Environment Water & Agriculture, Riyadh 11195, Saudi Arabia

**Keywords:** lice, acari, essential gene, drug targets, vaccine candidates, subtractive analysis

## Abstract

The emergence of drug-resistant lice, acari, and their associated pathogens (APs) is associated with economic losses; thus, it is essential to find new appropriate therapeutic approaches. In the present study, a subtractive proteomics approach was used to predict suitable therapeutics against these vectors and their infectious agents. We found 9701 proteins in the lice (*Pediculus humanus* var. *corporis*) and acari (*Ixodes scapularis*, *Leptotrombidium deliense*), and 4822 proteins in the proteomes of their APs (*Babesia microti*, *Borreliella mayonii*, *Borrelia miyamotoi*, *Borrelia recurrentis*, *Rickettsia prowazekii*, *Orientia tsutsugamushi* str. Boryong) that were non-homologous to host proteins. Among these non-homologous proteins, 365 proteins of lice and acari, and 630 proteins of APs, were predicted as essential proteins. Twelve unique essential proteins were predicted to be involved in four unique metabolic pathways of lice and acari, and 103 unique proteins were found to be involved in 75 unique metabolic pathways of APs. The sub cellular localization analysis of 115 unique essential proteins of lice and acari and their APs revealed that 61 proteins were cytoplasmic, 42 as membrane-bound proteins and 12 proteins with multiple localization. The druggability analysis of the identified 73 cytoplasmic and multiple localization essential proteins revealed 22 druggable targets and 51 novel drug targets that participate in unique pathways of lice and acari and their APs. Further, the predicted 42 membrane bound proteins could be potential vaccine candidates. Screening of useful inhibitors against these novel targets may result in finding novel compounds efficient for the control of these parasites.

## 1. Introduction

Vector-borne pathogens endure in nature by implementing various arthropods as hosts. Several of these agents have been found increasing in distribution to novel areas due to global warming and anthropogenic events have endorsed promising environments for the persistence and proliferation of these agents. Anthropogenic events include agriculture practices, community sports and leisure, globalization of travel and trade, and forest invasion, which ease human exposure to these agents proliferated in the modified natural environment [1,2,3].

Mites and ticks are two evolutionarily linked dissimilar assemblies of arthropods belonging to the class Arachnida and subclass Acari. The nourishing habits of mites and ticks are quite distinct from each other. Mites show diverse feeding manners as herbivores, predators, blood, and keratin feeding parasites. Ticks, on the other hand, are obligatory blood-feeding organisms of several vertebrates except for fishes. Plant damage caused by herbivorous mites might result in lower agricultural yields [4]. Predatory mites can be employed to reduce herbivorous mites, but parasitic mites and ticks cause skin irritations, stress, and decreased production of meat, milk, wool, and leather. These vectors and vectors-borne parasites are harmful to humans and animals health [5,6,7,8,9].

Lice (Anoplura) are wingless, hematophagous insects infecting mammals and birds [10,11]. Among mammals, humans have been known as a favored host for *Pediculus humanus* and *Pthirus pubis* (pubic lice). There are two morphotypes of *P. humanus*: *P. humanus* var. *capitis* (head lice) and *P. humanus* var. *corporis* (body lice) [12]. The occurrence of body louse has been related to poverty and their transmission occurs in refugee camps and homeless shelters, particularly in the unhygienic environment [13,14]. *Borrelia recurrentis* (louse-borne relapsing fever) and *Rickettsia prowazekii* are the common harmful bacteria transmitted by body louse (epidemic typhus) [15]. On the other hand, ticks are notorious ectoparasites transmitting diversified infectious agents than other groups of blood-feeding arthropods [16,17,18]. Tick-borne diseases cause thousands of human and animal deaths resulting in significant morbidities. The tick species belonging to the genus *Ixodes* are vectors of numerous infectious agents of multiple medical and veterinary importance. Blacklegged tick, *Ixodes scapularis* infestation outcomes in the proliferate of many pathogens like the agents that cause Lyme disease (*Borreliella burgdorferi* sensu stricto and *Borreliella mayonii*) babesiosis (*Babesia microti*, *Babesia odocoilei*), anaplasmosis (*Anaplasma phagocytophilum*), *Borrelia miyamotoi* disease (*B. miyamotoi*), an utmost health problem of the livestock sector [19,20,21,22,23,24,25,26,27].

The available proteomic and genomic sequences of pathogens have been assistive in the discovery and categorization of novel therapeutic targets and vaccine candidates. The subtractive proteomics analysis is an in silico approach for the prediction of genes that are extremely conserved, essential for the reproduction and existence of microbial pathogens [28,29,30]. These conserved genes are the major part of metabolic pathways for the survival of infectious agents having no resemblance to the genes of the host. Inhibiting these conserved genes, which are required for a variety of biological processes, would be deadly for the pathogens. These non-host homologous vital genes could serve as the prospective potential drug targets for the upcoming drug development stage. Therefore, the subtractive proteomics approach may significantly decrease the time needed to characterize prospective therapeutics targets.

Our previous report revealed the relevance of the prediction of drug and vaccines candidates using the subtractive proteomics approach against ticks and tick-borne pathogens [30]. However, the data on drug targets and vaccine candidates were limited to ticks and tick-borne pathogens. In this study, non-host homologous, differential pathway analysis, subcellular localization prediction, and uncharacterized proteins functional family classification of the proteome of lice, acari, and their associated pathogens was done to predict potential therapeutic targets for the prevention of these disease-causing agents.

## 2. Materials and Methods

### 2.1. Proteomes Retrieval of Vectors, Host, and Pathogens

Lice (*Pediculus humanus* var. *corporis*) and Acari (*Ixodes scapularis*, *Leptotrombidium deliense*), their associated pathogens (*Babesia microti*, *Borreliella mayonii*, *Borrelia miyamotoi*, *Borrelia recurrentis*, *Rickettsia prowazekii*, *Orientia tsutsugamushi* str. Boryong), and host (*Homo sapiens*) proteome were downloaded from NCBI (National Center for Biotechnological Information). The selection of the lice, acari, and their associated pathogens was based on their available proteome, KEGG pathways (Kyoto Encyclopedia of Genes and Genomes pathway database), and the KAAS (KEGG Automatic Annotation Server) list for their KO (KEGG Orthology). Proteins with less than 100 AA residues were excluded from the analysis [31].

### 2.2. Removal of Duplicate Sequences 

To pinpoint the paralogs within the proteome of lice, acari, and their APs, CD-HIT Suite was used at 60% to cleansed proteomes [30,32]. These proteins were eliminated, and the non-paralogous proteins were chosen for additional analysis.

### 2.3. Non-Host Homologous Proteins

For non-paralogous proteins, BLASTp analysis was performed against the proteomes of *H. sapiens*, using an E-value cut-off of 10^−5^ [33]. Non-host homologous proteins were chosen for further study after proteins exhibiting homologous characters with the hosts (*H. sapiens*) were removed. 

### 2.4. Identification of Essential Proteins 

To find genes necessary for lice, acari, and their acari-borne pathogens (APs), non-host homologous proteins were BLASTp analyzed against the Database of Essential Genes (DEG) with an E-value cut-off score of < E 10^−10^. A 100 bit-score of minimums was used to choose essential proteins of selected lice, acari, and their APs.

### 2.5. Analysis of Metabolic Pathway 

KAAS and KEGG were used to predict the essential proteins in the metabolic pathways [34]. The unique pathways in the lice, acari, and APs were predicted after comparing their metabolic pathways with their host metabolic pathways (*H. sapiens*). Those metabolic pathways were selected as unique pathways exclusive to pathogens and not found in the host. Further, the essential proteins were searched in the unique pathways and enlisted unique proteins having a role in unique pathways.

### 2.6. Druggability Evaluation of Essential and Unique Proteins

The essential and unique proteins found inside the unique pathways of lice, acari, and their APs were BLASTp against the DrugBank database containing the Food and Drug Administration (FDA) approved drug targets [35]. Essential and unique proteins that showed matched frequencies against the approved drugs database of FDA were considered the druggable targets. Further, essential and unique proteins that did not reveal a matching hit with the approved drugs of the FDA were considered to be novel targets.

### 2.7. Subcellular Localization 

The PSORTb v.3.0. tool has been frequently used for predicting the cellular location of proteins [36]. The PSORTb predicted different subcellular localization for unique essential proteins found in the cytoplasm, cytoplasmic membrane, cell membrane, and extracellular as well as proteins having unknown location.

### 2.8. Virulence of Non-Host Homologous Unique Target Proteins

The virulency of unique target proteins was determined using the Virulence Factors Database (VFDB) [37]. The proteins were BLASTp searched against a database of protein sequences from the VFDB core dataset (R1) with a cut-off bit-score > 100 and an E-value of 10^−5^.

### 2.9. Protein Functional Family Classification

The SVMProt web server was used to classify uncharacterized essential target proteins into functional families [38]. For the protein classification into a functional family, the SVMProt operates a Support Vector Machine (SVM) that utilizes the primary protein sequence data.

### 2.10. Protein–Protein Interaction of Selected Membrane Proteins

The STRING database (http://string-db.org (accessed on 8 January 2020)) was used to determine the most promising metabolic functional connections among all identified proteins [39]. The STRING database provides a critical evaluation as well as the additional interactions of protein–protein, involving associations directly and indirectly.

### 2.11. B-Cell Linear Epitopes Prediction

B-Cell linear epitopes prediction was performed using three algorithms: BCPred [40] with default epitope length of 14 amino acids reporting only non-overlapping epitopes above 75% specificity, ABCPred [41] with an epitope length of 14 amino acids reporting only non-overlapping epitopes above the 0.8 thresholds, and BepiPred [42] with the 0.5 minimal thresholds and at least 6 aa identified as epitopes.

**Figure 1:**
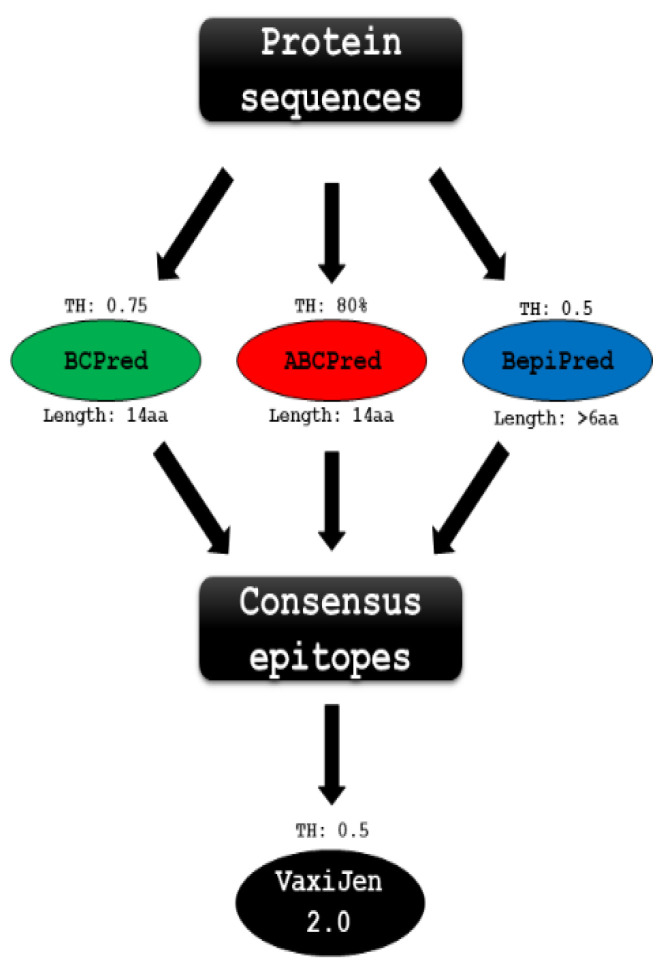
Flowchart of B-cell linear epitope prediction.

Amino acid sequences that were identified as probable epitopes by at least two algorithms were subjected to subsequent analysis in the VaxiJen v.2.0 [43]. VaxiJen allows the classification of antigens based on their physicochemical features. The threshold of 0.5 was used, which is the default recommendation for analyzing the sequence of parasite proteins (Figure 1).

### 2.12. D Structure Prediction

The three-dimensional model of proteins was predicted using the I-TASSER composite process [44]. This tool generates its models through a threading technique, which considers protein folding rather than similarities. The models with the highest confidence scores were submitted to ModRefiner [45] for subsequent refinement.

### 2.13. Discontinuous B-Cell Epitope Prediction 

Using the two-prediction tool, three-dimensional models were evaluated for the existence of discontinuous epitopes. The ElliPro [46] tool utilizes Thornton’s method together with residue clustering algorithms. The parameters recommended by the tool were used to predict the epitopes with more than 6 amino acids (minimum score 0.5 and maximum distance 6). The DiscoTope method [47] uses calculations to determine the surface accessibility of epitope fragments; it used the standard threshold (−3.7) in this analysis.

## 3. Results and Discussions

The vector–host–pathogen proteome has been mined using subtractive proteomics to suggest possible new therapeutic targets and vaccination candidates. The subtractive proteomics approach has been shown as a potential method for the identification of unique potential drug targets against numerous pathogens [28,29,30]. In this study, we aimed to predict the vital novel drug targets and vaccine candidates as alternate potential therapeutics against lice, acari, and their APs. The complete proteomes of lice (*P. humanus* var. *corporis*), acari (*I. scapularis*, *L. deliense*), and their APs (*B. microti*, *B. mayonii*, *B. miyamotoi*, *B. recurrentis*, *R. prowazekii*, *O. tsutsugamushi* str. Boryong) retrieved from the NCBI database are comprised of 10,775, 20,467, 14,667, 3601, 1133, 1118, 1012, 843, and 1085 proteins in their proteome, respectively. *Babesia odocoilei* transmitted by *Ixodes scapularis* was not included in this study due to the unavailability of genome and proteome for this pathogen. After removal of the proteins having amino acids less than 100, the dataset was submitted to the CD-HIT program at 60% identity [48]. The retrieved non-redundant dataset was comprised of 14,618 proteins for *I. scapularis* while 9726, 11,328, 3363, 922, 906, 890, 772, and 731 proteins for *P. humanus* var. *corporis*, *L. deliense*, *B. microti*, *B. mayonii*, *B. miyamotoi*, *B. recurrentis*, *R. prowazekii*, and *O. tsutsugamushi* str. Boryong, respectively. The non-redundant dataset was submitted to BLASTp against the host to filter the proteins that did not have any resemblance to the host proteins (*H. sapiens*) [30,49]. Out of 14,618 *I. scapularis* proteins, 5619 proteins were found to be non-homologous to *H. sapiens* while 2210, 1872, 1780, 704, 696, 677, 492, and 473 proteins of *P. humanus* var. *corporis*, *L. deliense*, *B. microti*, *B. mayonii*, *B. miyamotoi*, *B. recurrentis*, *R. prowazekii* str. Madrid E, and *O. tsutsugamushi* str. Boryong, respectively, were found to be non-host homologous (*H. sapiens*) proteins (Table 1 and Table 2).

**Figure 2:**
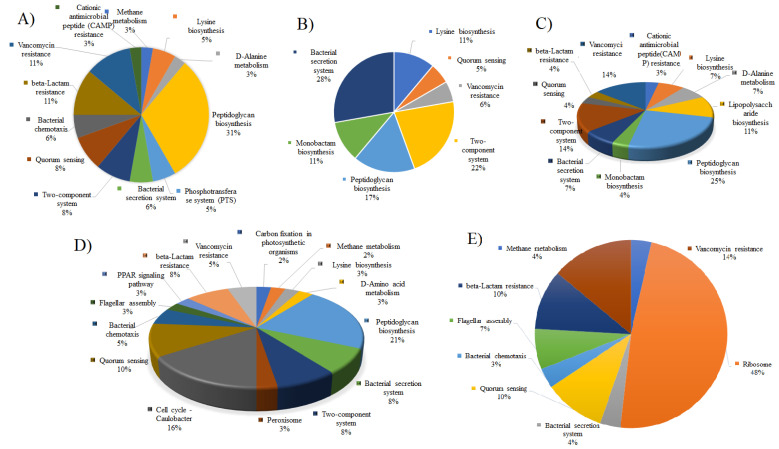
Unique non-homologous essential proteins in the unique metabolic pathway of (**A**) *Borrelia recurrentis*, (**B**) *Orientia tsutsugamushi* str. Boryong, (**C**) *Rickettsia prowazekii* str. Madrid E, (**D**) *Borreliella mayonii*, and (**E**) *Borrelia miyamotoi*.

These non-homologous sets of proteins were BLASTp against the Database of Essential Gene (DEG) [50] with 60% identity, an E-value cut-off score of 10^−5^, and a bit-value greater than 100 [30]. This set included required for the pathogen’s survival. Thus, 169 proteins were identified as essential proteins for *I. scapularis* while 120, 76, 106, 95, 121, 119, 95, and 94 were essential proteins in the case *P. humanus* var. *corporis*, *L. deliense*, *B. microti*, *B. mayonii*, *B. miyamotoi*, *B. recurrentis*, *R. prowazekii*, and *O. tsutsugamushi* str. Boryong, respectively. The comparison was conducted between metabolic pathways of acari and APs with the pathways of the host (*H. sapiens*) to enlist unique metabolic pathways present inside the KEGG Database. The obtained result showed that 79 pathways are unique to acari and APs, while the rest of the pathways were common in acari and APS, and their host (*H. sapiens*). These 79 unique pathways possess 115 essential proteins, except *B. microti* which has no protein involved in these unique pathways. Some of these 115 proteins were predicted to be involved in several unique pathways (Figure 2A–E). Such proteins are crucial for survival as they are an integral component of the response that generates or utilizes unique substrate materials, distinctively found in lice, acari, and their APs. Therefore, these 115 target proteins were chosen as final targets for further analysis, except *B. microti* which has no target protein (Supplementary Tables S1 and S2). In the case of *B. microti*, we found 27 essential targets that were pathways independent and can be further analysed for therapeutic targets as this study was limited to the pathways dependent target proteins (Supplementary Table S3). Further, the reannotation of the *B. microti* genome and KEGG pathways may assist in predicting target proteins against this infectious agent.

**Figure 3:**
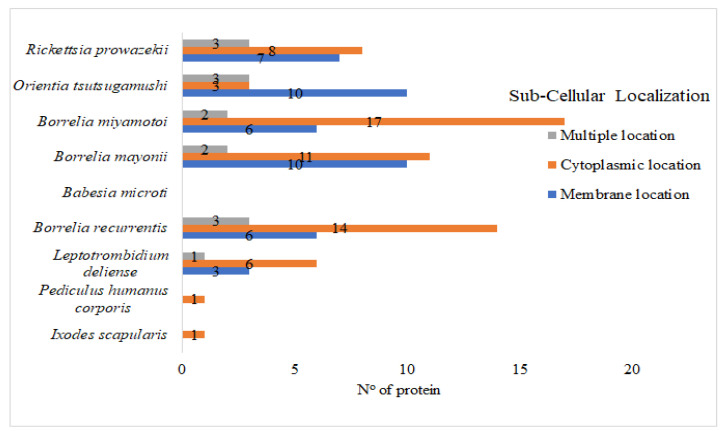
Cellular localization of the essential protein in lice, acari, and their associated pathogens.

The PSORTb server was used to look for cytoplasmic proteins that could be pharmacological targets and outer membrane proteins that could be potential vaccine candidates among the 115 target proteins [30,36]. The subcellular localization of 115 target proteins was predicted to be 61 in the cytoplasm, 42 in the membrane, and 12 with multiple localization (Figure 3, Supplementary Tables S1 and S2). Small molecule drug development can consider cytoplasmic proteins, while vaccine development can involve membrane or secreted proteins [51]. To assess any possible druggability, 73 target proteins were BLASTp against the dataset proteins of FDA-approved drugs [35]. Among them, 22 proteins hit the DrugBank database (Supplementary Table S1). Moreover, the druggability analysis revealed 51 proteins that do not hit the DrugBank database, thus considered to be novel drug targets (Supplementary Table S1).

Screening for Virulence Factors (VFs) has proven to be a promising method for predicting therapeutic targets [37,52]. To locate virulence, the 115 unique target proteins were BLASTp against the core dataset (R1) of VFDB. The results showed that out of 115 target proteins, 29 proteins were virulent, and 86 proteins were non-virulent (Supplementary Tables S1 and S2). The suppression of virulence proteins could make the disease causing agents non-infectious, hence may be used as potential drug targets and vaccine candidates [53].

The pathogen’s non-homologous essential proteins are prospective potential therapeutic targets and vaccine candidates [30,54]. Mining and filtration of identified proteins may help to shorten the time, labor, and resources required to develop therapeutic agents, as well as improve the chances of finding a good medicine and/or vaccination against pathogens [30,54]. Hence, the identified target proteins were described using additional factors that determine the suitability of therapeutic targets and vaccination candidates. Further, protein functional family prediction of the hypothetical proteins provides essential information regarding composition, activity, and metabolism [55]. Hypothetical proteins have an essential role in cellular networks, cell signaling, ions transport, the metabolite, as well as other molecules. Because they are involved in significant natural biological processes within a cell, they represent a large number of potential therapeutic targets [29,30,54].

The predicted 115 essential target proteins consisted of four putative uncharacterized proteins. Protein family classification permits uncharacterized proteins to be assigned a likely function [55]. Using the SVMProt online server, the four putative uncharacterized proteins were described and categorized. The result revealed one as a lipid-binding protein, two as a transport protein, and one protein as a transmembrane transporter. These transmembrane and transporter proteins could be useful therapeutic targets [55]. Information on disease-specific target proteins and metabolites involved in various metabolic pathways might help researchers find new treatment targets and understand how they interact with other molecules [56,57].

The peptidoglycan layer of bacteria is important in pathogenesis, because it helps the pathogen withstand osmotic lysis thus play an essential role in pathogenesis. Drug targets that inhibit peptidoglycan biosynthesis can reduce bacteria-generated infectivity [58]. MurA, MurC, MurD, MurE, and MurF are ADP making ligases that catalyze the addition of l-alanine, d-glutamate, a diamino acid, and d-alanine-d-alanine to UDP Nacetylmuramic acid in the biosynthesis of peptidoglycan [58]. In this study, 21 proteins including UDP-N-acetylglucosamine, 1-carboxyvinyltransferase, UDP-N-acetylmuramoyl-l-alanyl-d-glutamate--2,6-diaminopimelate ligase, undecaprenyl-diphosphate phosphatase, UDP-N-acetylmuramoyl-l-alanine--d-glutamate ligase, phospho-N-acetylmuramoyl-pentapeptide-transferase, UDP-N-acetylmuramoyl-tripeptide--d-alanyl-d-alanine ligase, BP1A family penicillin-binding protein, undecaprenyldiphospho-muramoylpentapeptide beta-N-acetylglucosaminyltransferase, UDP-N-acetylmuramate--l-alanine ligase, PASTA domain-containing protein, and UDP-N-acetylmuramate dehydrogenase were found to be essential for *B. recurrentis*. The UDP-N-acetylmuramate--l-alanine ligase, NUDP-N-acetylmuramate--l-alanine ligase, undecaprenyldiphospho-muramoylpentapeptide beta-N- acetylglucosaminyltransferase, penicillin binding protein (pbpA2), UDP-N-acetylglucosamine 1-carboxyvinyltransferase, phospho-N-acetylmuramoyl-pentapeptide-transferase, and UDP-N-acetylmuramoylalanyl-d-glutamate--2,6-diaminopimelate ligase were found to be essential for *R. prowazekii* Madrid E. The UDP-N-acetylglucosamine 1-carboxyvinyltransferase, phospho-N-acetylmuramoyl-pentapeptide-transferase, UDP-N-acetylmuramate dehydrogenase were found to be essential in *O. tsutsugamushi* str. Boryong. The UDP-N-acetylglucosamine 1-carboxyvinyltransferase, phospho-N-acetylmuramoyl-pentapeptide-transferase, undecaprenyl-diphosphate phosphatase, UDP-N-acetylmuramoyl-l-alanine--d-glutamate ligase, UDP-N-acetylmuramoyl-l-alanyl-d-glutamate--2,6-diaminopimelate ligase, UDP-N-acetylmuramate dehydrogenase, undecaprenyldiphospho-muramoylpentapeptide beta-N-acetylglucosaminyltransferase, and transpeptidase family protein were found to be essential in *B. mayonii*. These 29 proteins have been identified as being involved in APs’ unique pathways, and could potentially be used as therapeutic targets.

The two-component system is a signal transduction system that is responsible for detecting any alteration in the environment or within the bacterial cell state and is crucial for bacteria development and survival in extreme conditions [59]. Three proteins such as chromosomal replication initiator protein DnaA, response regulator, and chemotaxis protein CheB were found to be essential for *B. recurrentis*. Four proteins, PETR protein (ompR), osmolarity sensor protein ENVZ (envZ), cytochrome D ubiquinol oxidase subunit II (cydB), and chromosomal replication initiation protein were found to be essential for *R. prowazekii* Madrid E. Four proteins including chromosomal replication initiator protein DnaA, response regulator transcription factor, PleD family two-component system response regulator, and two-component sensor histidine kinase in *O. tsutsugamushi* str. Boryong were found to be essential proteins, and chromosomal replication initiator protein DnaA, response regulator and methyl-accepting chemotaxis protein in *B. mayonii* were found throughout this pathway. As a result, targeting these fourteen proteins could impair pathways essential for APs survival and might be potential therapeutic targets.

The phosphoenolpyruvate carbohydrate phosphotransferase system (PTS) is limited solely to bacteria and plays a key role in catalyzing the transportation and phosphorylation of sugars and their derivatives [60]. The sucrose-specific IIBC component of the PTS system could be one of two key targets. Their hydrophilic B domains were exposed at the outer membrane, but their hydrophobic C domain was entrenched in the plasma membrane, allowing them to prevent glucose uptake and phosphorylation [61]. These two proteins have the potential to be used as therapeutic targets.

The remaining 70 essential target proteins were found to be essential in the insect hormone biosynthesis pathway, D-Alanine metabolism pathway, lysine biosynthesis pathway, bacterial chemotaxis pathway, methane metabolism, quorum sensing pathway, bacterial secretion system pathway, monobactam biosynthesis pathway, lipopolysaccharide biosynthesis pathway, beta-Lactam resistance pathway, vancomycin resistance pathway, cationic antimicrobial peptide (CAMP) resistance pathway, Ribosome pathway, Carbon fixation in photosynthetic organisms pathway, D-Amino acid metabolism pathway, Peroxisome pathway, Cell cycle—Caulobacter pathway, Flagellar assembly pathway, and PPAR signaling pathway (Supplementary Table S4). As a result, targeting these 115 proteins could disrupt pathways important for lice, acari, and their APs survival, making them viable therapeutic targets.

After the prioritization of the final 115 target proteins, 42 proteins were found in the membrane, hence considered as vaccine candidates (Supplementary Table S2) [30]. The antigenicity of membrane proteins revealed that 26 proteins out of 42 were the most potent antigenic proteins, with the highest antigenic prediction score greater than 0.6. Out of 42 membrane proteins, only 12 proteins revealed a virulence factor (VFs). Finally, only nine membrane proteins were found to be involved in the virulence as well as essential targets (Supplementary Table S2). These proteins, which have a variety of functions, may be better suited for the development of vaccine candidates since they reduce virulence and are required for ABP survival.

**Figure 4:**
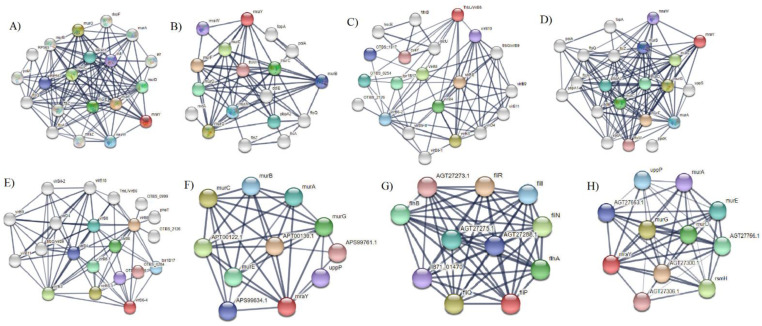
Protein–protein interaction of protein using string database. Nodes of the network represent protein while protein–protein interaction are presented by edges. The red colors show the query proteins and first shell of interaction, and the white nodes show the second level of interaction. Empty nodes show proteins of unknown 3D structure, and filled nodes show proteins of known or predicted 3D structure. (**A**) phospho-N-acetylmuramoyl-pentapeptide-transferase membrane proteins (*Rickettsia prowazekii* str. Madrid E). (**B**) UDP-N-acetylenolpyruvoylglucosamine reductase membrane proteins of *Orientia tsutsugamushi* str. Boryong. (**C**) conjugal transfer protein membrane proteins of *O. tsutsugamushi* str. Boryong. (**D**) Phospho-N-acetylmuramoyl-pentapeptide-transferase membrane proteins of *O. tsutsugamushi* str. Boryong. (**E**) Type IV secretion system protein membrane proteins of *O. tsutsugamushi* str. Boryong. (**F**) phospho-N-acetylmuramoyl-pentapeptide-transferase of *Borrelia miyamotoi*, (**G**) flagellar type III secretion system pore protein FliP of *B. miyamotoi*, and (**H**) phospho-N-acetylmuramoyl-pentapeptide-transferase of *Borreliella mayonii*.

Nine membrane proteins having a role in virulence and essential targets were used individually as a query for the string database to predict protein–protein interactions [39]. Eight proteins were found to have a network in the string database and each protein showed more than 10 interactions with other proteins inside the network. Interactors with a confidence score greater than or equal to 0.700 were set as a criterion in the protein network, and the target protein with more interactors was considered as metabolically active which could be an appropriate therapeutic target [62,63] (Figure 4A–H). 

Formulations based on epitopes are currently considered one of the main strategies in vaccine development due to their cost and time benefit in process optimization [64]. One of the main advantages of this method is the ability to focus the immune response towards crucial epitopes, avoiding the production of antibodies against regions of no interest [65]. Using in silico linear and conformational-based epitope prediction algorithms, the ten membrane proteins shown to play a role in virulence b-cell epitopes were identified (Figure 1). Analyzes performed in linear epitope prediction algorithms (ABCPred, BCPred, BepiPred) indicated consensus sequences in all evaluated proteins (Supplementary Figure S1). 

**Figure 5:**
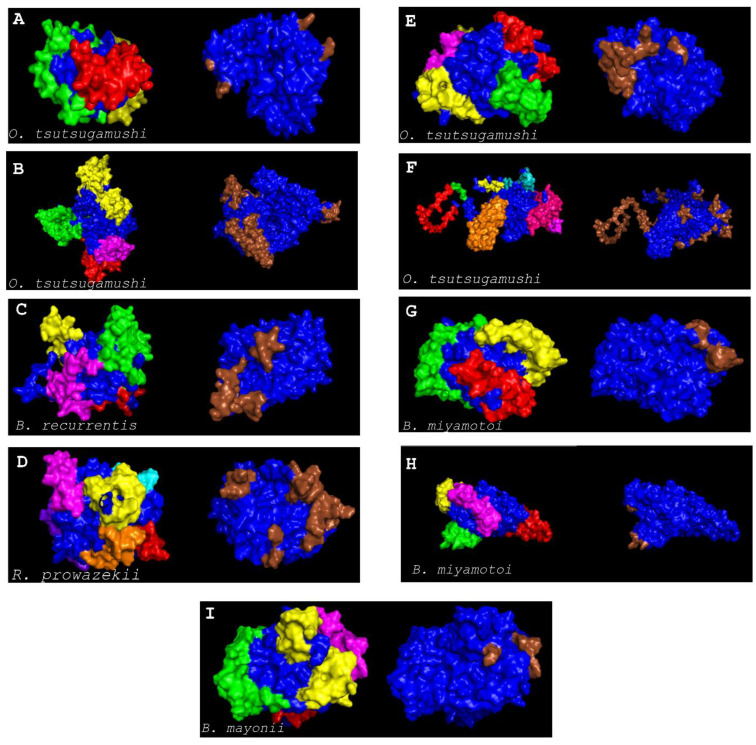
The three-dimensional structure of the protein was predicted by I-TASSER and Discontinuous epitope prediction of the putative epitopes identified by ElliPro (left and with colored epitopes) and DiscoTope (right and epitopes in brown). (**A**) UDP-N-acetylmuramate dehydrogenase (*Orientia tsutsugamushi*; WP_011944569.1), (**B**) Type IV secretion system protein (*O. tsutsugamushi*; WP_011945117.1), (**C**) Phospho-N-acetylmuramoyl-pentapeptide-transferase (*Borrelia recurrentis*; WP_012538808.1), (**D**) Phospho-N-acetylmuramoyl-pentapeptide-transferase (*Rickettsia prowazekii* str. Madrid E; NP_220963.1), (**E**) Phospho-N-acetylmuramoyl-pentapeptide-transferase (*O. tsutsugamushi*; WP_011944610.1), (**F**) Type IV secretion system protein (*O. tsutsugamushi*; WP_011944382.1), (**G**) phospho-N-acetylmuramoyl-pentapeptide-transferase (*Borrelia miyamotoi*; WP_020954693.1), (**H**) flagellar type III secretion system pore protein FliP (*B. miyamotoi*; WP_020954665.1) and (**I**) phospho-N-acetylmuramoyl-pentapeptide-transferase (*Borreliella mayonii*; WP_075552002.1).

Three-dimensional structures of the nine selected proteins were predicted using the I-TASSER and refined with ModRefiner tools (Figure 5). Moreover, discontinuous epitope analysis (ElliPro and DiscoTope) indicated the presence of likely sequences of interest in all targets (Supplementary Figure S2). The prediction data showed that continuous epitopes are related to discontinuous epitopes. Moreover, the results showed that it is important to use multiple prediction tools, since the combination of the linear and conformational predictions improves the results [66].

## 4. Conclusions

In this study, we employed a subtractive proteomics approach to identify and characterize potential essential proteins that could be exploited as effective therapeutic targets and vaccine candidates to reduce infectious diseases caused by lice, acari, and their APs. The retrieval of available proteomes of lice (*P. humanus* var. *corporis*), acari (*I. scapularis*, *L. deliense*), and their APs (*B. microti*, *B. mayonii*, *B. miyamotoi*, *B. recurrentis*, *R. prowazekii* and *O. tsutsugamushi* str. Boryong) assisted to shortlist proteins based on their non-redundant, non-host homologous, essentiality, virulence factor, and druggability. The 115 proteins were shortlisted as therapeutic targets. Only 10 proteins were identified as potential vaccine candidates, followed by B-cell linear epitopes prediction. The development of novel therapeutics against disease-causing agents could be aided by screening these targets. Experimental validation of these drug targets and vaccine candidates by immunological, biochemical, and molecular approaches may assist in the effective control of infectious agents.

## Figures and Tables

**Table 1:** The stepwise analysis and their result of selected vectors.

Steps	*Ixodes scapularis*	*Pediculus humanus* var. *corporis*	*Leptotrombidium deliense*
		Host	
	*Homo sapiens*	*Homo sapiens*	*Homo sapiens*
Total proteome	20,467	10,775	14,667
Duplicates (>60% identical) in CD-HIT	14,618	9726	11,328
Non-homologs	5619	2210	1872
Essential proteins in DEG	169	120	76
Unique metabolic pathway KEGG	1	3	-
Essential Proteins involved KEGG and KAAS	1	1	10
Druggability with cutoff E-value 10^−5^	0	0	1

**Table 2:** Subtractive proteome analysis and metabolic pathway results of lice, acari, and their associated pathogens.

Steps	*Babesia microti* str. R1	*Borreliella mayonii*	*Borrelia miyamotoi*	*Borrelia recurrentis*	*Rickettsia prowazekii* str. Madrid E	*Orientia tsutsugamushi* str. Boryong
			Host			
	*Homo sapiens*	*Homo sapiens*	*Homo sapiens*	*Homo sapiens*	*Homo sapiens*	*Homo sapiens*
Total proteome	3601	1133	1118	1012	843	1085
Duplicates (>60% identical) in CD-HIT	3363	922	906	890	772	731
Non-homologs	1780	704	696	677	492	473
Essential proteins in DEG	106	95	121	119	95	94
Unique metabolic pathway KEGG	3	15	8	16	21	12
Essential Proteins involved KEGG and KAAS	-	23	25	23	18	14
Druggability with cutoff E-value 10^−5^	-	5	8	6	6	3

## Data Availability

The data that support the findings were derived from the following resources available in the public domain (GenBank) at https://www.ncbi.nlm.nih.gov/genbank/ (accessed on 30 April 2019).

## Supplementary Materials

The following are available online at https://www.mdpi.com/article/10.3390/vaccines10010008/s1, Supplementary Table S1. Characteristic features of pathway-based novel and druggable target proteins. Supplementary Table S2. Characteristic features of pathway-based vaccine candidates. Supplementary Table S3. Non-homologous essential proteins involved in *Babesia microti* pathways independent targets and their KO list. Supplementary Table S4. Essential proteins involved in lice, acari specific pathway, their KO list, and their pathways. Supplementary Figure S1. Linear epitope prediction of the vaccine candidates. The putative epitopes above the threshold were predicted by BCPred (green boxes), ABCPred (red boxes) and BepiPred (blue boxes). Putative epitopes recognized by at least two predictive tools (bold letters) were submitted to VaxiJen. Finally, the sequences with the highest VaxiJen score were selected as putative parasite epitopes (black background and bold letters); phospho-N-acetylmuramoyl-pentapeptide-transferase (*Borrelia recurrentis*; WP_012538808.1); phospho-N-acetylmuramoyl-pentapeptide-transferase (*Rickettsia prowazekii* str. Madrid E; NP_220963.1); type IV secretion system protein (*Orientia tsutsugamushi*; WP_011945117.1); type IV secretion system protein (*O. tsutsugamushi*; WP_011944382.1); phospho-N-acetylmuramoyl-pentapeptide-transferase (*O. tsutsugamushi*; WP_011944610.1) and UDP-N-acetylmuramate dehydrogenase (*O. tsutsugamushi*; WP_011944569.1); phospho-N-acetylmuramoyl-pentapeptide-transferase (*Borrelia miyamotoi*; WP_020954693.1); flagellar type III secretion system pore protein FliP (*B. miyamotoi*; WP_020954665.1) and phospho-N-acetylmuramoyl-pentapeptide-transferase (*Borreliella mayonii*; WP_075552002.1). Supplementary Figure S2. Discontinuous epitope prediction of the putative epitopes deduced by ElliPro (left and with colored epitopes) and DiscoTope (right and epitopes in brown). The amino acid sequences and detailed epitopes identified by ElliPro (colored letters) and DiscoTope (underlined and bold letters). (A) UDP-N-acetylmuramate dehydrogenase (*Orientia tsutsugamushi*; WP_011944569.1) (B) Type IV secretion system protein (*O. tsutsugamushi*; WP_011945117.1), (C) Phospho-N-acetylmuramoyl-pentapeptide-transferase (*Borrelia recurrentis*; WP_012538808.1), (D) Phospho-N-acetylmuramoyl-pentapeptide-transferase (*Rickettsia prowazekii* str. Madrid E; NP_220963.1), (E) Phospho-N-acetylmuramoyl-pentapeptide-transferase (*O. tsutsugamushi*; WP_011944610.1), (F) Type IV secretion system protein (*O. tsutsugamushi*; WP_011944382.1), (G) phospho-N-acetylmuramoyl-pentapeptide-transferase (*Borrelia miyamotoi*; WP_020954693.1); (H) flagellar type III secretion system pore protein FliP (*B. miyamotoi*; WP_020954665.1) and (I) phospho-N-acetylmuramoyl-pentapeptide-transferase (*Borreliella mayonii*; WP_075552002.1).
