# Supplementary material for: Prediction of Novel Drug Targets and Vaccine Candidates against Human Lice (Insecta), Acari (Arachnida), and Their Associated Pathogens
Source: Vaccines (Basel). 2021 Dec 22;10(1):8. doi: 10.3390/vaccines10010008 (PMC8778234; doi:10.3390/vaccines10010008)
Supplement: Supplementary file 1 [file vaccines-10-00008-s001.zip › Supplementary Figure S1.pdf]

A

MSTVISLPKINGEYRKNFKLSQLTWFKVGGISQVFYKPKDEEDLSCFLKNLQFDIQITVLGAGSNLLIRDNGIDGVTIKLGR
SFNEINFVKNNHYNIISVGAGTLNYDVAKFCLQHNLGGLEFLVGIPGTIGGGIAMNAGAYGQEFKDVVYSVEALDRLGNKHI
FLSKDLNFEYRQCIVNGFLIFTKTNLICYNDSKTSISQKLQKIQTVRKLTQPINQKTAGSAFRNTNNYKAWQLIDKVGLRGH
SIGGAQVSNLHCNFLINNGNATASDIENLGELIRKNVFDHTGITLEWEIKIVGKKSL

WP_011944569 UDP-N-acetylmuramate dehydrogenase [*Orientia tsutsugamushi*]

B

MTKILKILITKNKSLMTYYINILILLSYIFVSTSIQASNELLQAGEQLPTSNSLQWMDKYPNDNNTGCTNVSPTWLSVADVK
WIDIQANSNNNWIDSGIMTKAGKSISVEIPKISQNFRKLQKRYLVLHRVDPRFPVIGKTFIIELGTNNKPISRLHNFENGKL
LNYQNENSSNFQNGSTNFTNAINLQKKFFNGADTKISVKAGDIIDIALISSVDFFNKLQLKGGTEKSGFTGELYPRWDDYKY
YKPYGIYTVSLKSNVGVVDNALLVTGQTSDKKALSKLLVGVKSIDSISSFEKCNLNNLSKSQSCIMQSGIGMEIKLDQEVLK
SKFDKFLVGYNVGNANSSAFLDKPIEGMYHIVAKSDGDLSFSTPLFNNNVKYRTNGLQDVEYSSILSSCSTLNEFEQKYLNS
ILSSKQEIIQGIVVDKTLVGRYLMYITIDRHNIVSDEYDGDIEYIISNTTPNLSTKGTTLPRNGINIVTPESAKLWFRFVKT
NNEQFNLKVTVPPDSEGKIKVAGFFYDNIYIPIKQKVEKFSKLFYFGLAKNAALKKVFSILAILYITLYGIYFLLGVVKVTA
YDLLIRCSKIIVIAALFNESSQYIFYDTLFPMFDGGINSLISYAVKTTASDVDNPFKFFDLVVSRYIDVNFLKIILIEIVNI
HNGLTILGILTLWSIMRFIIIMVKVCMELLMSMIAIAILVGLAPMFIIFILFDRTAEIFKRWLTALLLNTLMPVIMIIFILI
INELMLVAVEAAFPEIRICWGTLFDIELNLDLSAIGLPTAFSIPLMAVPFYNVVFVGGNLFNAMDLGNSFAGSLAGVFLLYN
LVLLAGTLVGSQVFTKGINRKGMSYVKSIMMQLLS

WP_011945117 type IV secretion system protein [*Orientia tsutsugamushi*]

C

MFCFLGLRLLKYITFRTAYATIFAFLLALIFGPFIISRLKKLKLDQILRKDGPKHHLSEKMGIPTMGGVLIFFCVLVSLFFW
IHFFNIYFLIVLFVMVSFACLGFTDDLLKIKRKNSDGLNPKFKIYGQILFSFISVVMLYYFGGEHVSILYFPFFKSLKLDLG
ILYIPFGMFVLISASNSFNLTDGLDGLAIGLSIVVIGALIIIAYLTSRVDFALYLNIPNVKGCEELVIFLGALLGGSFGFLW
FNAYPAKIMMGDTGSLSIGAVLGMVALILKSEILFAILAGVFVVETLSVIIQVVVYKKTKKRVFKMAPLHHHFEELGWSEMQ
VVIRFWIIGLIFAILALSTIKIR

WP_012538808 phospho-N-acetylmuramoyl-pentapeptide-transferase [*Borrelia recurrentis*]

D

MLYNLLLPHIHNSHIANLFHYITFRSGLAIIITLSISFVTGPILIKFLRSLQKYGQPIRSDGPESHKTKAGTPTMGGIMIIL
SSCLSTLLLADLTNKYIWITLFGFISFGIIGFMDDYAKVKRNNHYGVRGKSKFLLQGIISLIIYVLLEYLDKNFSHLLNVPF
FKNLSLDLNYFYMVFAIFVIVGSSNAVNLTDGLDGLATVPIAFTAGSFALISYLVGNLIYANYLQLTYIPNTGELTVLCAGL
VGSCLGFLWFNAQPAEVFMGDTGSLSLGGVLGIISVITKHEIVLAIIGGLFVIETTSVILQVYYFKATKGKRIFKMAPLHHH
FEKHGWAESKVVIRFWIISVIFSLIGLSSLKLR

NP_220963 phospho-N-acetylmuramoyl-pentapeptide-transferase [*Rickettsia prowazekii*]

E

MLYNLLVSHINSCYISNIFYNVIVRSGIAILLSFSISFSLIPILIKYFKYWKNLAQPIRNLGHKSHIAKAGTPTMGGIAIVF
SIIISTLMLADYKNIYVLTTIFVMLSLAILGLIDDYQKVTKKNTKGINATYKLISQIMVSVICCMIVNYNLDSEIANHLIIP
FFKKLTIDLSIFYIPFALFIIIGSSNAVNLTDGLDGLVTVPIIIVAFCLGLMCYLADNAQYININHLQILHVQQASELTVLC
SAIIGASLGFLWYNIQPAKIFMGDVGSLSLGGAIGIISVISKNEIRLGIIGGLFVIEALSAIIQIYSIRYLGGKRVFKMAPI
HHHFEQIGWSESKIVSRFWLLSIIFSLIGLSSLIL

WP_011944610 phospho-N-acetylmuramoyl-pentapeptide-transferase [*Orientia tsutsugamushi*]

F

MILHNFSEFNTRNLNIMNQQNTLFLKFLVGIILFCSVISYSIAECIDADDFGFPIIAISSRYDTKQLTGQKDNQVAPWIDSK
LLVNGKPLVVMVKHWNYHEYDNDISHLSAWSAWYGTNKNKHTLASITKRFPECRFRNNKTFSDSYDDNDDIPVINPPCLFKH
GIGLYALIAKPGVDPNANVHSQSYGIPKKTINFHVGQNYLSSLNSTELDSGFLDTTPDGNIVKTGGYFHKYQDQEAEQYVGG
RLYFKILDRFYDDNNGQYKIIIKSGVGDEKDSPETFLINIVKEMLFGNKKNQNKNGIIQNLFINILKNPSYKIVVNLTLILF
IAFSGLAFLIGNINMTAHELVLRTVKILVISVLLNSDTAWKFFYDYLFFIFVDGPQFIIKTINEATAIGPGSSSILGLMIAP
HTLKKLFSILFVDWGGFIYIICYLILLYYIFIISFKATVLYLNALILVGIGIIVGPVFLCFVLFQFTKPIFENWIKQLTIYA
LQPVILFAGIAFVGMFIRHEIYASLGFRVCEVPFPPIANTLIKIISGDSSKKQSLLNLWFPAQVLKKTLLFSQKCANIPVPE
DHIVYRDQKPWQCSDGISGNSKQLITSTDPSNEKHCSAYECKANRYVELPFLDPNINKDRYRIRNFFAGNFVQWDSLLLLAA
CVFLLSMFNDNAIALANYISSGGDRSSASEKSTTAIVSTVTPPSPTTEIPAAFSKIGQQRTKNSNSHSNSNSRSGINTGPKK

WP_011944382.1 type IV secretion system protein [*Orientia tsutsugamushi*]

G

MFYLLGLRLLQYITFRTAYATIFAFLFSLILGPFIIVRLKKLKLDQILREDGPRRHLSAKTGIPTMGGILIFFCVLVSLFFW
INPWNIYFLIILFVMISFACLGFIDDFLKIKRKNSDGLNPSLKIYGQVFFSCISVTMLYYFGGDHVSIIYFPFFKSLKLDLG
VLYIPFGIFILISASNSFNLTDGLDGLAIGLSIVVTGSLVIIAYLTSRADFAFYLNIPNIKGSEELVVFLGALLGGSFGFLW
FNAYPAKIMMGDTGSLSIGAILGMVALILKSEILFAILTGVFIVETLSVIIQVAVYKKTKKRVFRMAPLHHHFEELGWSEMQ
VVIRFWIIGLIFAILALSTLKIR

WP_020954693 phospho-N-acetylmuramoyl-pentapeptide-transferase [*Borrelia miyamotoi*]

H

MNKKLSLFLFFGAMNFLFAQTKSLQPTNGLNFPFVDFVNSGGSGIIFPLQLLLILTIITLSPAFLVLMTSFLRIAIVLDFIR
RALSLQQSPPNQIIMGLALFLTIFTMWPTFNIIYEDAYLPLKESKIGFNQFYDKGIAPLRNFMYKQMSNSRHEEIRLFMKIS
NYSRPKNFSEVPTHVLIASFILHELKIAFKMGILIFLPFIVIDIVVSAVLMAMGMIMLPPVMISLPFKLILFVMVDGWTLIT
SGLVKSFM

WP_020954665 flagellar type III secretion system pore protein FliP [*Borrelia miyamotoi*]

MFYLLGLRLLKYITFRMAYATIFAFLLSLIVGPYVILRLKKLRADQILREDGPKRHLSEKAGIPTMGGILIFFCVFISLVFW
SNILNVYFLIMVFVMLGFAFLGFIDDFL**KIKNKTSDGLK**ARFKIYGQIIFSFISVGILYYFGSEHVSIIYFPFIKSFQIDLG
LFYIPFGMFILISASNSFNLTDGLDGLAIGLSIVITGALIIIAYLTSRADFAAYLYIPNIKGSEELVIFLGALLGGSFGFLW
FNAYPAKIMMGDTGSLALGAILGMAALILKSEILFSILAGVFIIETMSVII**QVLVYKKTKKRVFK**MAPLHHHFEELGWSEMQ
VVIRFWIIGLIFAIIALSTIKIR

WP_075552002 phospho-N-acetylmuramoyl-pentapeptide-transferase [*Borreliella mayonii*]
