# Supplementary material for: Prediction of Novel Drug Targets and Vaccine Candidates against Human Lice (Insecta), Acari (Arachnida), and Their Associated Pathogens
Source: Vaccines (Basel). 2021 Dec 22;10(1):8. doi: 10.3390/vaccines10010008 (PMC8778234; doi:10.3390/vaccines10010008)
Supplement: Supplementary file 1 [file vaccines-10-00008-s001.zip › Supplementary Figure S2.pdf]

A

WP_011944569.1 UDP-N-acetylmuramate dehydrogenase [*Orientia tsutsugamushi*]

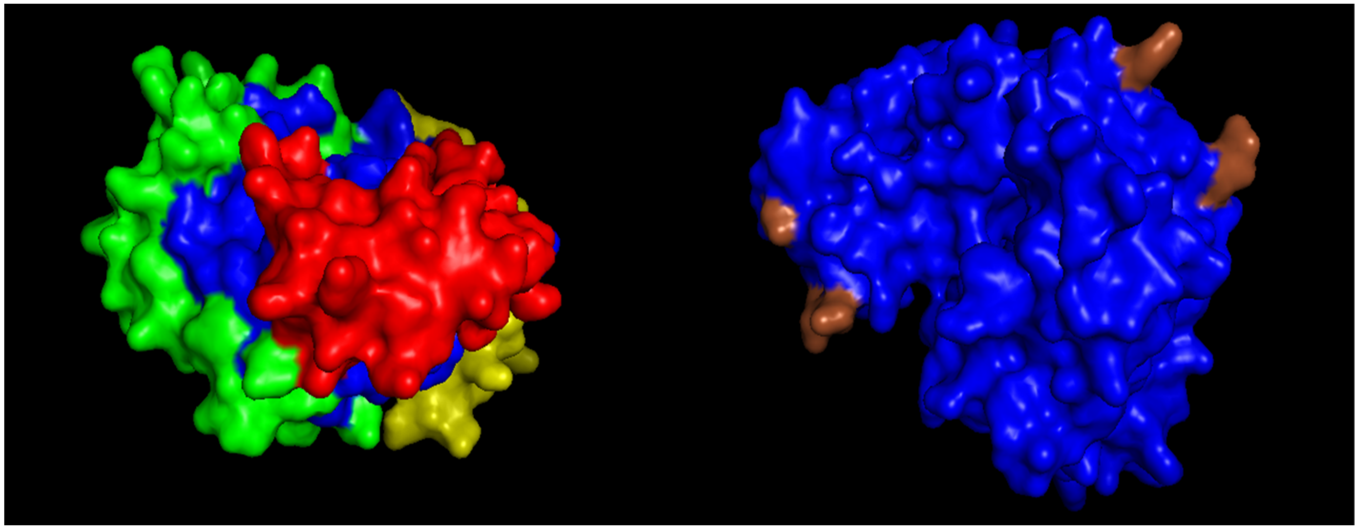

MSTVISLPKI**N**GEYRKNF**K**LSQLTWFKVGGISQVFYKPKDEEDLSCFLKNLQFDIQITVLG
AGSNLLIRDNGIDGVTIKLGRSFNEINFVKNNHYNIISVGAGTLNYDVAKFCLQHNLGGLE
FLVGIPGTIGGGIAMNAGAYGQEFKDVVYSVEALDRLGNKHIFLSKDLNFEYRQCIVNGFL
IFTKTNLICYNDSK**T**SISQKLQKIQTVRK**L**TQPINQKTAGSAFRNT**NN**YKAWQLIDKVGLR
GHSIGGAQVSNLHCNFLINNGNATASDIENLGELIRKNVFDHT**G**ITLEWEIKIVGKKSL

B

WP_011945117.1 type IV secretion system protein [*Orientia tsutsugamushi*]

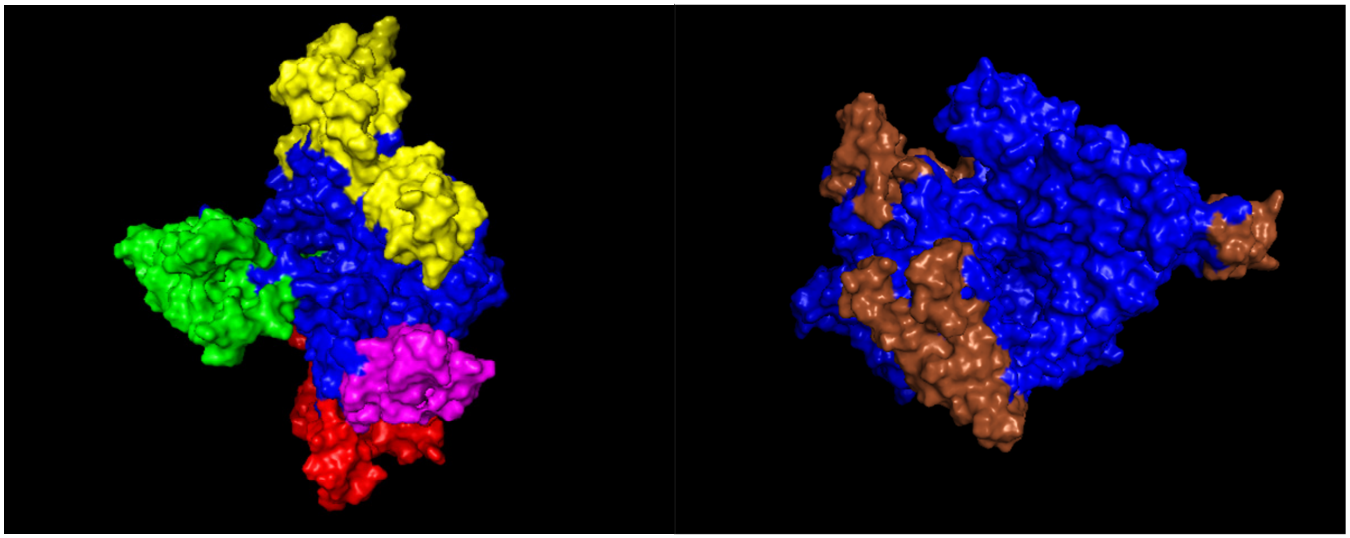

MTKILKILITKNKSLMTYYINILILLSYIFVSTSIQASNELLQAGEQLPTSNSLQWMDKYPN
DNNTGCTNVSPTWLSVADVKWIDIQANSNNNWIDSGIMTKAGKSISVEIPKISQNFRKLQKR
YLVLHRVDPRFPVIGKTFIIELGTNNKPISRLHNFENGKLLNYQNENSSNFQNGSTNFTNAI
NLQKKFFNGADTKISVKAGDIIDIALISSVDFFNKLQLKGGTEKSGFTGELYPRWDDYKYYK
PYGIYTVSLKSNVGVVDNALLVTGQTSDKKALSKLLVGVKSIDSISSFEKCNLNNLSKSQSC
IMQSGIGMEIKLDQEVLKSKFDKFLVGYNVGNANSSAFLDKPIEGMYHIVAKSDGDLSFSTP
LFNNNVKYRTNGLQDVEYSSILSSCSTLNEFEQKYLNSILSSKQEIIQGIVVDKTLVGRYLM
YITIDRHNIVSDEYDGDIEYIISNTTPNLSTKGTTLPRNGINIVTPESAKLWFRFVKTNNEQ
FNLKVTVPPDSEGKIKVAGFFYDNIYIPIKQKVEKFSKLFYFGLAKNAALKKVFSILAILYI
TLYGIYFLLGVVKVTAYDLLIRCSKIIVIAALFNESSQYIFYDTLFPMFDGGINSLISYAVK
TTASDVDNPFKFFDLVVSRYIDVNFLKIILIEIVNIHNGLTILGILTLWSIMRFIIIMVKVC
MELLMSMIAIAILVGLAPMFIIFILFDRTAEIFKRWLTALLLNTLMPVIMIIFILIINELML
VAVEAAFPEIRICWGTLFDIELNLDLSAIGLPTAFSIPLMAVPFYNVVFVGGNLFNAMDLGN
SFAGSLAGVFLLYNLVLLAGTLVGSQVFTKGINRKGMSYVKSIMMQLLS

C WP_012538808.1 phospho-N-acetylmuramoyl-pentapeptide-transferase [*Borrelia recurrentis*]

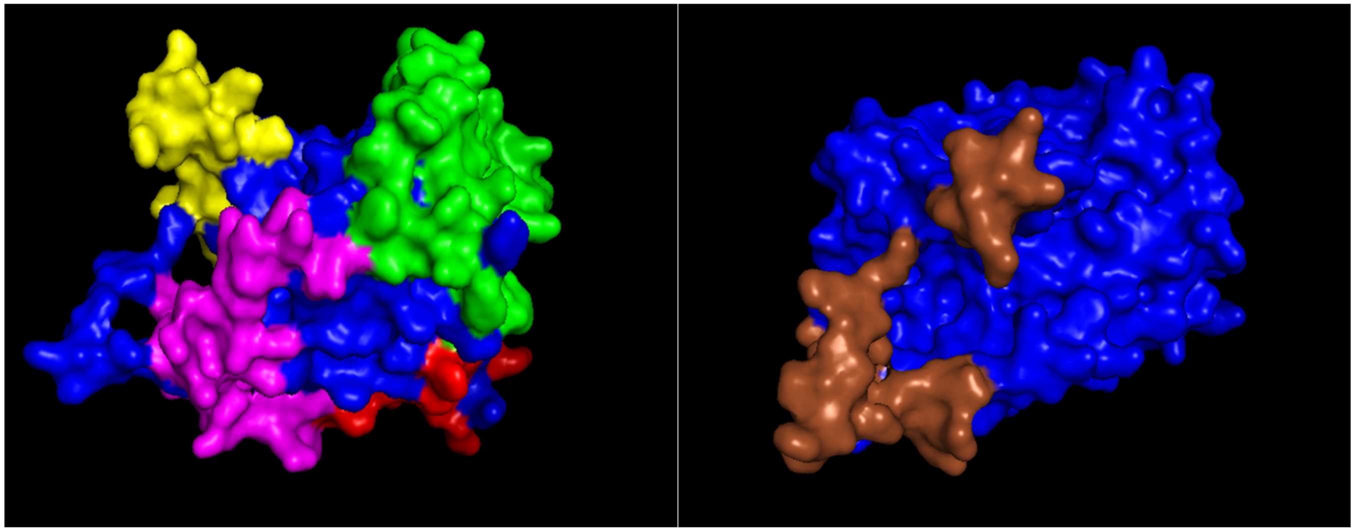

MFCFLGLRLLKYITFRTAYATIFAFLLALIFGPFIISRLKKLK**L**DQ**IL****RKDGPKHHLSE****K**M
GIPTMGGVLIFFCVLVSLFFWIHFFNIYFLIVLFVMVSFACLGFTDDLLKI**KRKN**SDGLNP
KFKIYGQILFSFISVVMLYYFGGEHVSILYFPFFKSLKLDLGILYIPFGMFVLISASNSFN
LTDGLDGLAIGLSIVVIGALIIIAYLTSRVDFALYLNIPNVKGCEELVIFLGALLGGSFGF
LWFNAYPAKIMMGDTGSLSIGAVLGMVALILKSEILFAILAGVFVVETLSVIIQVVVYK**KT**
KKRVFKMAPLHHHFEELGWSEMQVVIRFWIIGLIFAILALSTIKIR

D

NP_220963.1 phospho-N-acetylmuramoyl-pentapeptide-transferase [*Rickettsia prowazekii* str. Madrid E]

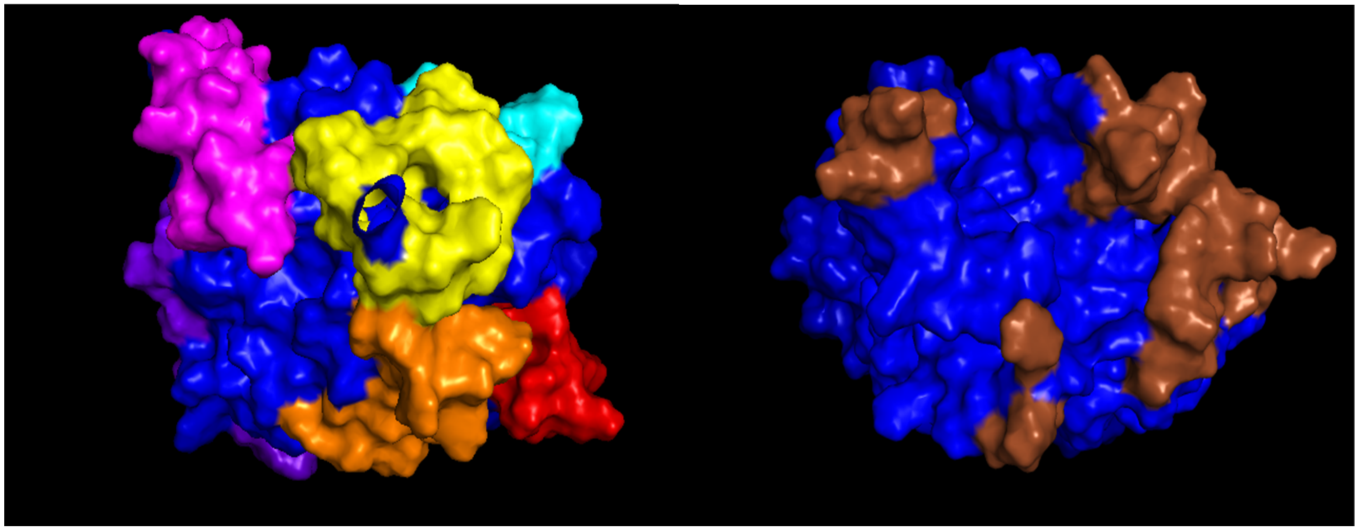

MLYNLLLPHIHNSHIANLFHYITFRSGLAIIITLSISFVTGPILIKFLRSLQKYGQPIRSD
GPESHKTKAGTPTMGGIMIILSSCLSTLLLADLTNKYIWITLFGFISFGIIGFMDDYAKVK
RNNHYGVRGKSKFLLQGIISLIIYVLLEYLDKNFSHLLNVPFFKNLSLDLNYFYMVFAIFV
IVGSSNAVNLTDGLDGLATVPIAFTAGSFALISYLVGNLIYANYLQLTYIPNTGELTVLCA
GLVGSCLGFLWFNAQPAEVFMGDTGSLSLGGVLGIISVITKHEIVLAIIGGLFVIETTSVI
LQVYYFKATKGKRIFKMAPLHHHFEKHGWAESKVVIRFWIISVIFSLIGLSSLKLR

E WP_011944610.1 phospho-N-acetylmuramoyl-pentapeptide-transferase [*Orientia tsutsugamushi*]

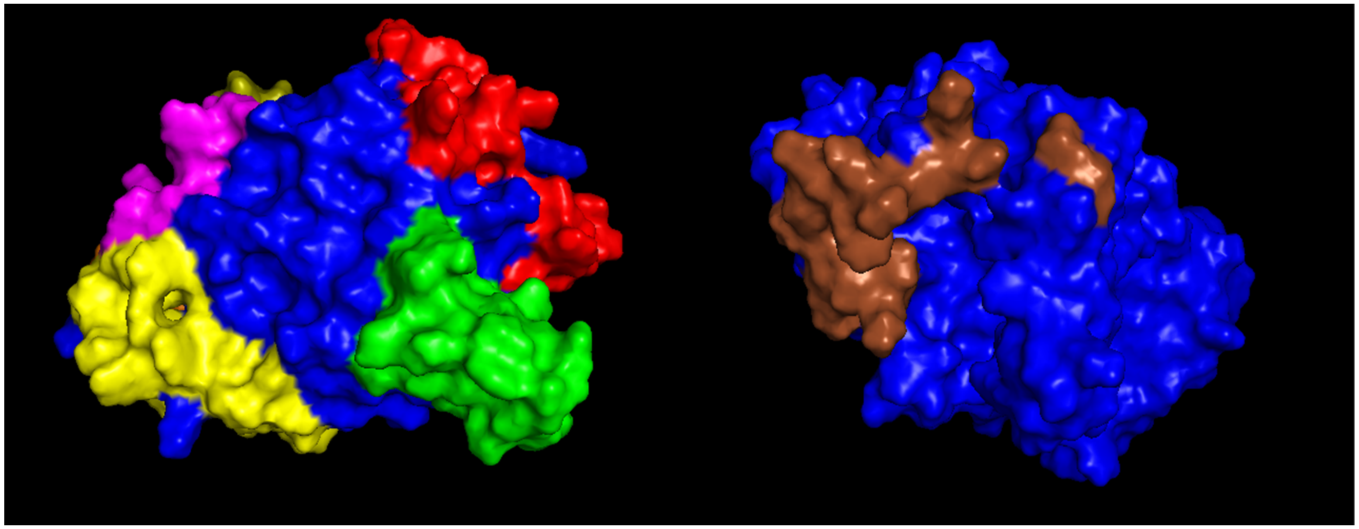

MLYNLLVSHINSCYISNIFYNVIVRSGIAILLSFSISFSLIPILIKYFKYWKNLAQ**PIRNL**
GHKSHIAKAGTPTMGGIAIVFSIIISTLMLADYKNIYVLTTIFVMLSLAILGLIDDYQKVT
KKNTKGINATYKLISQIMVSVICCMIVNYNLDSEIANHLIIPFFKKLTIDLSIFYIPFALF
IIIGSSNAVNLTDGLDGLVTVPIIIVAFCLGLMCYLADNAQYININHLQILHVQQASELTV
LCSAIIGASLGFLWYNIQPAKIFMGDVGSLSLGGAIGIISVISKNEIRLGIIGGLFVIEAL
SAIIQIYSIRYLGGKRVFKMAPIHHHFEQ**IG**WSESKIVSRFWLLSIIFSLIGLSSLIL

F

WP_011944382.1 type IV secretion system protein
[*Orientia tsutsugamushi*]

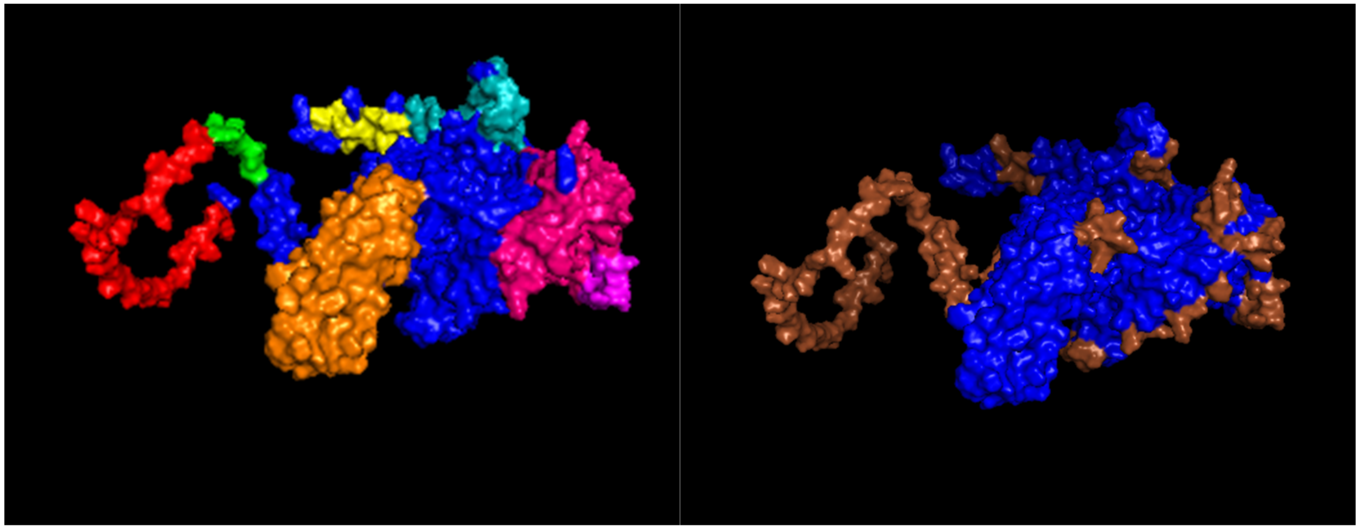

MILHNFSEFN**TRNLNIM**NQQNTLFLKFLVGIILFCSVISYSIAECIDADDFGFPIIAISSR
Y**DTKQ**L**TGQKDN**QVAPWIDSKLLVNGKPLVVMVKHWN**YHE**YDNDISHLSAWSAWYGT**NK**NK
HTLASITKRFPECRFRNNKTF**SDSYDDNDD**IPVINPPCLFKHGIGLYALIAKPGVDPNANV
HSQSYGI**PK**KTINFHVGQNYLSSLNSTELDSGFLDTTPDGNIVKTGGYFHKY**QDQ**EAEQYV
GGRLYFKILDRFYDDNNGQYKIIIKSGVGDEKDSPETFLINIVKEMLFG**NKKNQNKN**GIIQ
NLFINILKNPSYKIVVNLTLILFIAFSGLAFLIGNINMTAHELVLRTVKILVISVLLNSDT
AWKFFYDYLFFIFVDGPQFIIKTINEATAIGPGSSSILGLMIAPHTLKKLFSILFVDWGGF
IYIICYLILLYYIFIISFKATVLYLNALILVGIGIIVGPVFLCFVLFQFTKPIFENWIKQL
TIYALQPVILFAGIAFVGMFIRHEIYASLGFRVCEVPFPPIANTLIKIISGD**SSKK**QSLLN
LWFPAQVLKKTLLFSQKCANIPVPEDHIVYRDQKPWQCSDGISGNSKQLI**T**S**TDPS**NEKHC
S**AYECKANRYVELPFLDPNINKDRYRIRNFFAGNFVQWDSLLLLAACVFLLSMFNDNAIAL**
ANYISSGGDRSSASEKSTTAIVSTVTPPSPTTFIPAAFSKIGQQRTKNSNSHSNSNSRSGI
NTGPKK

G

WP_020954693.1 phospho-N-acetylmuramoyl-pentapeptide-transferase [*Borrelia miyamotoi*]

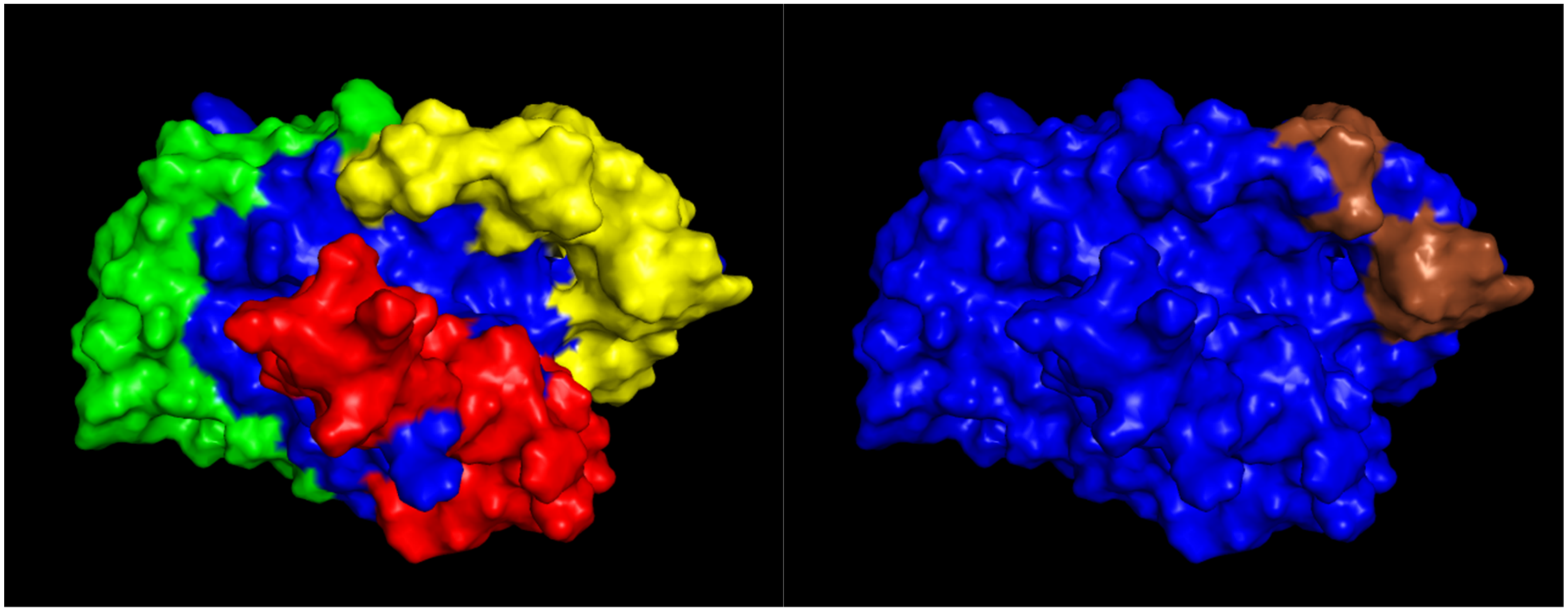

MFYLLGLRLLQYITFRTAYATIFAFLFSLILGPFIIVRLKKLKLDQILRE**DGPRRH**LSAKTGIPTM
GGILIFFCVLVSLFFWINPWNIYFLIILFVMISFACLGFIDDFLKIK**RKNS**DGLNPSLKIYGQVFF
SCISVTMLYYFGGDHVSIIYFPFFKSLKLDLGVLYIPFGIFILISASNSFNLTDGLDGLAIGLSIV
VTGSLVIIAYLTSRADFAFYLNIPNIKGSEELVVFLGALLGGSFGFLWFNAYPAKIMMGDTGSLSI
GAILGMVALILKSEILFAILTGVFIVETLSVIIQVAVYKKTKKRVFRMAPLHHHFEELGWSEMQVV
IRFWIIGLIFAILALSTLKIR

H WP_020954665.1 flagellar type III secretion system pore protein FliP [*Borrelia miyamotoi*]

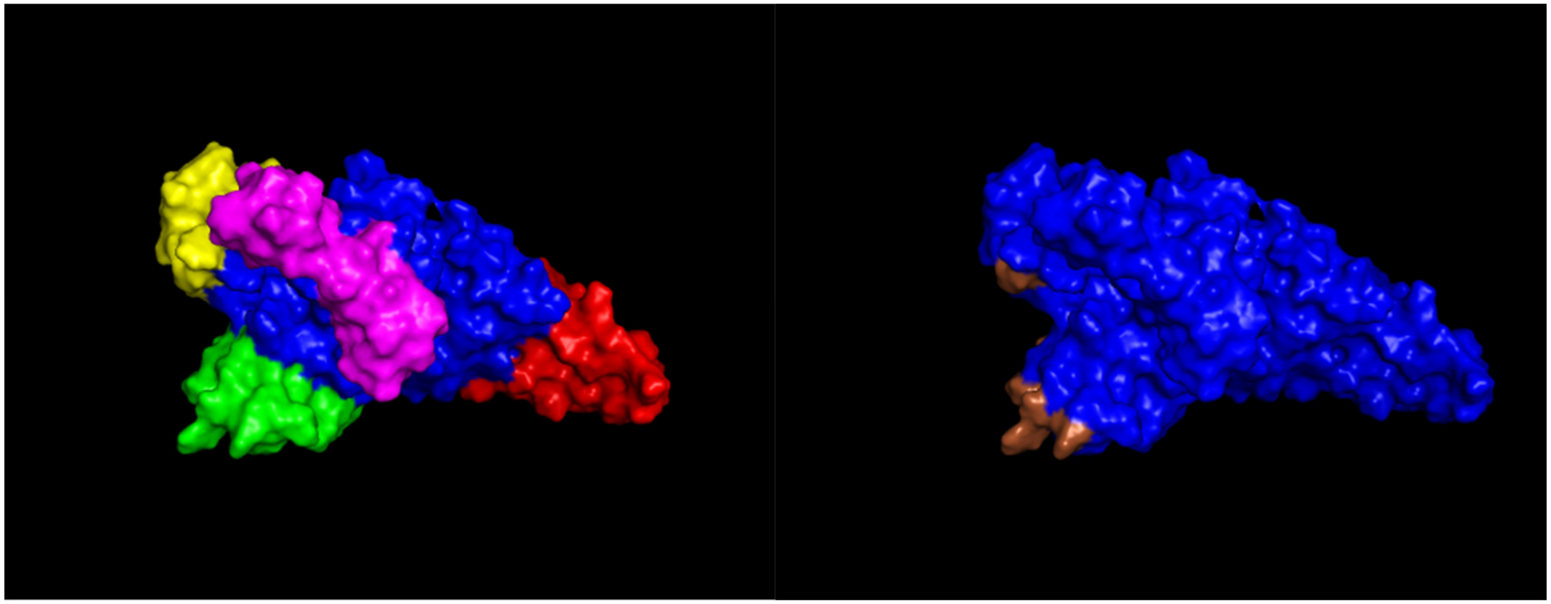

MNKKLSLFLFFGAMNFLFAQTKSLQPTNGLNFPFVDFVNSGGSGIIFPLQLLLILTIITLSPAFLVL
MTSFLRIAIVLDFIRRALSLQQSPPNQIIMGLALFLTIFTMWPTFNIIYEDAYLPLKESKIGF**N**QFY
DKGIAPLRNFMY**K**QM**S**NSRHEEIRLFMKIS**N**Y**SRPKN**FSEVPTHVLIASFILHELKIAFKMGILIFL
PFIVIDIVVSAVLMAMGMIMLPPVMISLPFKLILFVMVDGWTLITSGLVKSFM

WP_075552002.1 phospho-N-acetylmuramoyl-pentapeptide-transferase [*Borreliella mayonii*]

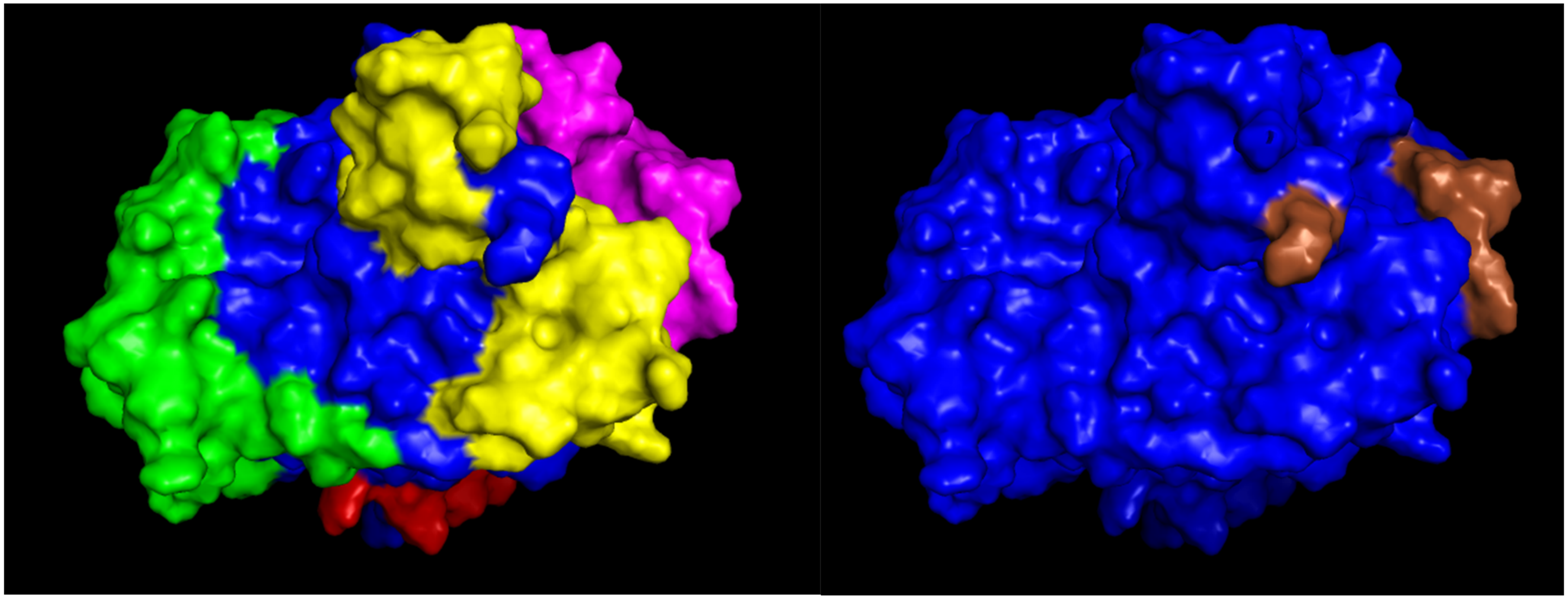

MFYLLGLRLLKYITFRMAYATIFAFLLSLIVGPYVILRLKKLRADQILRE**DGPKR**HLSEKAGIPTMGGIL
IFFCVFISLVFWSNILNVYFLIMVFVMLGFAFLGFIDDFLKIKNKTSDGLKARFKIYGQIIFSFISVGIL
YYFGSEHVSIIYFPFIKSFQIDLGLFYIPFGMFILISASNSFNLTDGLDGLAIGLSIVITGALIIIAYLT
SRADFAAYLYIPNIKGSEELVIFLGALLGGSFGFLWFNAYPAKIMMGDTGSLALGAILGMAALILKSEIL
FSILAGVFIIETMSVIIQVLVYKKTKKRVF**K**MAPLHHHFEELGWSEMQVVIRFWIIGLIFAIIALSTIKI
R
