# Supplementary material for: Prediction of Novel Drug Targets and Vaccine Candidates against Human Lice (Insecta), Acari (Arachnida), and Their Associated Pathogens
Source: Vaccines (Basel). 2021 Dec 22;10(1):8. doi: 10.3390/vaccines10010008 (PMC8778234; doi:10.3390/vaccines10010008)
Supplement: Supplementary file 1 [file vaccines-10-00008-s001.zip › Supplementary Table S1.pdf]

Supplementary Table S1. Characteristic features of pathway-based novel and druggable protein targets.

KEGG ID	NCBI Protein ID	Subcellular Location	Druggability	Virulence
isc00981	XP_002405220.1	Cytoplasmic	Novel	Non-virulent
phu00981	XP_002429994.1	Cytoplasmic	Novel	Non-virulent
bre00300	WP_012538729.1	Cytoplasmic	Novel	Non-virulent
bre02030	WP_012538886.1	Cytoplasmic	Novel	Virulent
bre02020	WP_012538902.1	Cytoplasmic	Novel	Non-virulent
bre00680	WP_012538908.1	Cytoplasmic	Novel	Non-virulent
bre00550	WP_012538809.1	Cytoplasmic	Novel	Non-virulent
bre02024	WP_012538824.1	Cytoplasmic	Novel	Virulent
bre02024	WP_012538906.1	Multiple location	Novel	Non-virulent
bre00550	WP_012539012.1	Cytoplasmic	Novel	Non-virulent
bre01503	WP_012539042.1	Multiple location	Novel	Virulent
bre02060	WP_012539046.1	Cytoplasmic	Novel	Non-virulent
bre00550	WP_012539148.1	Cytoplasmic	Novel	Non-virulent
bre00550	WP_012539190.1	Cytoplasmic	Novel	Non-virulent

rpr00261	NP_220539.1	Cytoplasmic	Novel	Non-virulent
rpr00300	NP_220965.1	Cytoplasmic	Novel	Non-virulent
rpr02020	NP_220969.1	Multiple location	Novel	Non-virulent
rpr00540	NP_221073.1	Cytoplasmic	Novel	Virulent
rpr00540	NP_220403.1	Cytoplasmic	Novel	Virulent
rpr00550	NP_220632.1	Cytoplasmic	Novel	Non-virulent
rpr00550	NP_220793.1	Multiple location	Novel	Non-virulent
rpr02020	NP_220807.1	Multiple location	Novel	Virulent
*	RWS22936.1	Cytoplasm	Novel	Non-virulent
*	RWS23662.1	Multiple location	Novel	Non-virulent
*	RWS26891.1	Cytoplasm	Novel	Non-virulent
*	RWS27540.1	Cytoplasm	Novel	Virulent
*	RWS28131.1	Cytoplasm	Novel	Non-virulent
*	RWS29218.1	Cytoplasm	Novel	Non-virulent
*	RWS29325.1	Cytoplasm	Novel	Non-virulent
ots02020	WP_011944869.1	Cytoplasm	Novel	Virulent
ots00300, ots00261	WP_011944488.1	Cytoplasm	Novel	Non-virulent

ots02020	WP_041621479.1	Multiple location	Novel	Virulent
bre00473	WP_012538701.1	Cytoplasmic	Druggable	Non-virulent
bre00550	WP_012538928.1	Cytoplasmic	Druggable	Non-virulent
bre02020	WP_012539000.1	Cytoplasmic	Druggable	Virulent
bre00550	WP_041178080.1	Multiple location	Druggable	Non-virulent
bre00550	WP_041178142.1	Cytoplasmic	Druggable	Non-virulent
rpr02020	NP_220808.1	Cytoplasmic	Druggable	Virulent
rpr00550	NP_220634.1	Cytoplasmic	Druggable	Non-virulent
rpr00550	NP_220950.1	Cytoplasmic	Druggable	Non-virulent
ott02020	WP_011944400.1	Cytoplasmic	Druggable	Virulent
bmo03010	WP_020954867.1	Multiple location	Druggable	Non-virulent
bmo01502	WP_043867855.1	Cytoplasm	Druggable	Non-virulent
bmo03010	WP_020954868.1	Cytoplasm	Novel	Non-virulent
bmo03010	WP_020954775.1	Multiple location	Novel	Non-virulent
bmo03010	WP_020954865.1	Cytoplasm	Novel	Non-virulent
bmo01502	WP_020954547.1	Cytoplasm	Druggable	Non-virulent

bmo03010	WP_020954864.1	Cytoplasm	Novel	Non-virulent
bmo00680	WP_020954819.1	Cytoplasm	Novel	Non-virulent
bmo03010	WP_020955140.1	Cytoplasm	Novel	Non-virulent
bmo02030	WP_020954921.1	Cytoplasm	Druggable	Virulent
bmo03010	WP_020954859.1	Cytoplasm	Druggable	Non-virulent
bmo03010	WP_020954869.1	Cytoplasm	Novel	Non-virulent
bmo01502	WP_020955128.1	Cytoplasm	Novel	Non-virulent
bmo03010	WP_020954871.1	Cytoplasm	Novel	Non-virulent
bmo03010	WP_020955059.1	Cytoplasm	Novel	Non-virulent
bmo03010	WP_020954773.1	Cytoplasm	Druggable	Non-virulent
bmo03010	WP_025443689.1	Cytoplasm	Druggable	Non-virulent
bmo02040	WP_172643126.1	Cytoplasm	Druggable	Virulent
bmo03010	WP_020954509.1	Cytoplasm	Novel	Non-virulent
bmay02020, bmay04112	WP_075552116.1	Cytoplasm	Novel	Non-virulent
bmay00550	WP_075552150.1	Cytoplasm	Druggable	Non-virulent
bmay04112	WP_075551834.1	Cytoplasm	Novel	Non-virulent

bmay02020, bmay02030	WP_075552215.1	Cytoplasm	Druggable	Virulent
bmay00710, bmay00680	WP_075552124.1	Cytoplasm	Novel	Non-virulent
bmay03070, bmay02024	WP_075551784.1	Multiple location	Novel	Non-virulent
bmay00300, bmay00550	WP_075551913.1	Multiple location	Novel	Non-virulent
bmay00550	WP_075552259.1	Cytoplasm	Druggable	Virulent
bmay04112	WP_075551999.1	Cytoplasm	Novel	Non-virulent
bmay02040	WP_075552359.1	Cytoplasm	Druggable	Virulent
bmay01501, bmay02024	WP_075552026.1	Cytoplasm	Novel	Non-virulent
bmay00550, bmay04112, bmay01502	WP_075552408.1	Cytoplasm	Novel	Non-virulent
bmay04112	WP_075551998.1	Cytoplasm	Druggable	Non-virulent

Note. *No KEGG organism name and pathways for *Leptotrombidium deliense*. We used BlastKoala to identify essential proteins in *L. deliense.*
