# Supplementary material for: Prediction of Novel Drug Targets and Vaccine Candidates against Human Lice (Insecta), Acari (Arachnida), and Their Associated Pathogens
Source: Vaccines (Basel). 2021 Dec 22;10(1):8. doi: 10.3390/vaccines10010008 (PMC8778234; doi:10.3390/vaccines10010008)
Supplement: Supplementary file 1 [file vaccines-10-00008-s001.zip › Supplementary Table S2.pdf]

Supplementary Table S2. Characteristic features of pathway-based vaccine candidates.

KEGG ID	NCBI Protein ID	Subcellular Location	Virulence	Antigenicity
bre03070	WP_012539063.1	Inner Membrane	Non-virulent	Antigenic
bre02060	WP_012539199.1	Inner Membrane	Non-virulent	Antigenic
bre00550	WP_012538779.1	Inner Membrane	Non-virulent	Antigenic
bre00550	WP_012538808.1	Inner Membrane	Virulent	Antigenic
bre02024	WP_025400666.1	Inner Membrane	Non-virulent	Non-antigenic
rpr02024	NP_221003.1	Inner Membrane	Non-virulent	Antigenic
rpr00540	NP_220482.1	Inner Membrane	Virulent	Non-antigenic
rpr03070	NP_220506.1	Inner Membrane	Non-virulent	Antigenic
rpr00550	NP_220963.1	Inner Membrane	Virulent	Antigenic
*	RWS21140.1	Inner Membrane	Non-virulent	Antigenic
*	RWS22251.1	Inner Membrane	Non-virulent	Antigenic
ots02024, ots03070	WP_011944421.1	Inner membrane	Non-virulent	Antigenic
ots01502, ots00550	WP_011944610.1	Inner membrane	Virulent	Antigenic
ots00300, ots00261	WP_011944930.1	Inner membrane	Non-virulent	Antigenic
ots02020	WP_011944228.1	Outer membrane	Non-virulent	Antigenic
ots03070	WP_011944753.1	Inner membrane	Non-virulent	Antigenic
ots03070	WP_011945113.1	Outer membrane	Virulent	Non-antigenic
ots03070	WP_011945117.1	Outer membrane	Virulent	Antigenic

ots03070	WP_011944382.1	Inner membrane	Virulent	Antigenic
bre00550	WP_012539122.1	Outer membrane	Non-virulent	Antigenic
rpr00473	NP_220488.1	Outer membrane	Non-virulent	Non-antigenic
rpr02020	NP_220603.1	Inner membrane	Non-virulent	Antigenic
rpr00550	NP_220939.1	Outer membrane	Non-virulent	Non-antigenic
*	RWS22448.1	Outer membrane	Non-virulent	Antigenic
ots00550	WP_011944970.1	Inner membrane	Non-virulent	Antigenic
ots00550	WP_011944569.1	Outer membrane	Virulent	Antigenic
bmo01502	WP_020954693.1	Inner membrane	Virulent	Antigenic
bmo02024, bmo03070	WP_020954873.1	Inner membrane	Non-virulent	Non-antigenic
bmo02024, bmo01501	WP_020954717.1	Outer membrane	Non-virulent	Non-antigenic
bmo02040	WP_020954665.1	Inner membrane	Virulent	Antigenic
bmo01501	WP_043867792.1	Outer membrane	Non-virulent	Non-antigenic
bmo01501, bmo02024	WP_020954720.1	Inner membrane	Non-virulent	Non-antigenic
bmay00550, bmay00470	WP_075552246.1	Outer membrane	Non-virulent	Non-antigenic
bmay00550, bmay01502	WP_075552002.1	Inner membrane	Virulent	Antigenic
bmay00550	WP_075551964.1	Inner membrane	Non-virulent	Antigenic
bmay04146	WP_075552339.1	Outer membrane	Non-virulent	Non-antigenic
bmay02030, bmay02024	WP_075552171.1	Inner membrane	Non-virulent	Antigenic
bmay03070	WP_075552309.1	Inner membrane	Non-virulent	Non-antigenic

bmay01501, bmay02024	WP_075552028.1	Inner membrane	Non-virulent	Non-antigenic
bmay00550, bmay01501	WP_075551856.1	Outer membrane	Non-virulent	Non-antigenic
bmay03320	WP_075552334.1	Outer membrane	Non-virulent	Non-antigenic
bmay02020, bmay02030	WP_075552331.1	Outer membrane	Virulent	Non-antigenic

Note. *No KEGG organism name and pathways for *Leptotrombidium deliense*. We used BlastKoala to identify essential proteins in *L. deliense.*
