# Supplementary material for: Prediction of Novel Drug Targets and Vaccine Candidates against Human Lice (Insecta), Acari (Arachnida), and Their Associated Pathogens
Source: Vaccines (Basel). 2021 Dec 22;10(1):8. doi: 10.3390/vaccines10010008 (PMC8778234; doi:10.3390/vaccines10010008)
Supplement: Supplementary file 1 [file vaccines-10-00008-s001.zip › Supplementry Table S3.pdf]

Supplementary Table S3. Non-homologous essential proteins involved in *Babesia microti* pathways independent targets and their KO list.

Accession no	Name of the protein	KO List
XP_012649607.1	2-C-methyl-D-erythritol 2,4-cyclodiphosphate synthase [*Babesia microti* strain RI]	K01770
XP_012649657.1	4-hydroxy-3-methylbut-2-enyl diphosphate reductase [*Babesia microti* strain RI]	K03527
YP_009363160.1	ribosomal protein L16 (apicoplast) [*Babesia microti* strain RI]	K02878
XP_012648738.2	Glutamine synthetase [*Babesia microti* strain RI]	K01915
XP_012650133.1	Elongation factor TS [*Babesia microti* strain RI]	K02357
XP_012650127.1	50S ribosomal protein L27 [*Babesia microti* strain RI]	K02899
XP_012650325.1	50S ribosomal protein L14 [*Babesia microti* strain RI]	K02874
XP_012648277.1	Chaperone protein DnaJ [*Babesia microti* strain RI]	K03686
XP_012647540.1	50S ribosomal protein L12, apicoplast, putative [*Babesia microti* strain RI]	K02935
XP_012648575.1	Aminomethyltransferase mitochondrial [*Babesia microti* strain RI]	K00605
XP_012649749.1	Probable nicotinate-nucleotide adenylyltransferase [*Babesia microti* strain RI]	K00969
XP_012650070.1	Elongation factor P [*Babesia microti* strain RI]	K02356
XP_021338311.1	Putative 50S ribosomal protein L18 apicoplast [*Babesia microti* strain RI]	K02881
XP_012649845.1	hypothetical protein BmR1_04g06200 [*Babesia microti* strain RI]	K02956
XP_021337917.1	Ribosomal protein L4/L1 family [*Babesia microti* strain RI]	K02926
XP_021338824.1	ATP synthase delta (OSCP) subunit [*Babesia microti* strain RI]	K02137
XP_021338233.1	solute carrier family 26 (sodium-independent sulfate anion transporter) [*Babesia microti* strain RI]	K03321

XP_021338688.1	AP endonuclease 1 [*Babesia microti* strain RI]	K01151
XP_021337866.1	tRNA pseudouridine synthase A 1 [*Babesia microti* strain RI]	K06173
XP_012650408.1	NOC2, nucleolar complex protein 2 [*Babesia microti* strain RI]	K14833
XP_012650060.1	Probable Bax inhibitor 1 [*Babesia microti* strain RI]	K21889
XP_021337899.1	F-type H+-transporting ATPase subunit delta [*Babesia microti* strain RI]	K02134
XP_021338308.1	ATP synthase (E/31 kDa) subunit [*Babesia microti* strain RI]	K02150
XP_012648988.1	4-diphosphocytidyl-2-C-methyl-D-erythritol kinase [*Babesia microti* strain RI]	K00919
XP_021338553.1	(E)-4-hydroxy-3-methylbut-2-enyl-diphosphate synthase [*Babesia microti* strain RI]	K03526
XP_021338244.1	6-phosphofructokinase [*Babesia microti* strain RI]	K24182
XP_012648489.1	Fumble [*Babesia microti* strain RI]	K24265
