# Supplementary material for: Prediction of Novel Drug Targets and Vaccine Candidates against Human Lice (Insecta), Acari (Arachnida), and Their Associated Pathogens
Source: Vaccines (Basel). 2021 Dec 22;10(1):8. doi: 10.3390/vaccines10010008 (PMC8778234; doi:10.3390/vaccines10010008)
Supplement: Supplementary file 1 [file vaccines-10-00008-s001.zip › Supplementry Table S4.pdf]

Supplementary Table S4. Essential proteins involved in lice, acarine specific pathway, their KO list, and their pathways.

**Pathways**	**Assigned KO list**	**Protein**	**Pathogen**
Two-component system	K02313	chromosomal replication initiator protein DnaA	*Borrelia recurrentis*
	K03413	response regulator	*Borrelia recurrentis*
	K03412	chemotaxis protein CheB	*Borrelia recurrentis*
	K07659	PETR protein (ompR)	*Rickettsia prowazekii* Madrid E
	K07638	osmolarity sensor protein ENVZ (envZ)	*Rickettsia prowazekii* Madrid E
	K00426	cytochrome D ubiquinol oxidase subunit II (cydB)	*Rickettsia prowazekii* Madrid E
	K02313	chromosomal replication initiation protein	*Rickettsia prowazekii* Madrid E
	K02313	chromosomal replication initiator protein DnaA	*Orientia tsutsugamushi* str. Boryong
	K13584	response regulator transcription factor	*Orientia tsutsugamushi* str. Boryong

	K02488	PleD family two-component system response regulator	*Orientia tsutsugamushi* str. Boryong
	K07716	two-component sensor histidine kinase	*Orientia tsutsugamushi* str. Boryong
	K02313	chromosomal replication initiator protein DnaA	*Borreliella mayonii*
	K03413	response regulator	*Borreliella mayonii*
	K03406	methyl-accepting chemotaxis protein	*Borreliella mayonii*
Peptidoglycan biosynthesis	K00790	UDP-N-acetylglucosamine 1-carboxyvinyltransferase	*Borrelia recurrentis*
	K01924	UDP-N-acetylmuramate--L-alanine ligase	*Rickettsia prowazekii* Madrid E
	K01921	D-alanine--D-alanine ligase	*Rickettsia prowazekii* Madrid E
	K02563	undecaprenyldiphospho-muramoylpentapeptide beta-N-acetylglucosaminyltransferase	*Rickettsia prowazekii* Madrid E
	K03587	penicillin binding protein (pbpA2)	*Rickettsia prowazekii* Madrid E
	K00790	UDP-N-acetylglucosamine 1-carboxyvinyltransferase	*Rickettsia prowazekii* Madrid E

	K01000	phospho-N-acetylmuramoyl-pentapeptide-transferase	*Rickettsia prowazekii* Madrid E
	K01928	UDP-N-acetylmuramoylalanyl-D-glutamate--2,6-diaminopimelate ligase	*Rickettsia prowazekii* Madrid E
	K01928	UDP-N-acetylmuramoyl-L-alanyl-D-glutamate--2,6-diaminopimelate ligase	*Borrelia recurrentis*
	K06153	undecaprenyl-diphosphate phosphatase	*Borrelia recurrentis*
	K01925	UDP-N-acetylmuramoyl-L-alanine--D-glutamate ligase	*Borrelia recurrentis*
	K01000	phospho-N-acetylmuramoyl-pentapeptide-transferase	*Borrelia recurrentis*
	K01929	UDP-N-acetylmuramoyl-tripeptide--D-alanyl-D-alanine ligase	*Borrelia recurrentis*
	K05366	BP1A family penicillin-binding protein	*Borrelia recurrentis*
	K02563	undecaprenyldiphospho-muramoylpentapeptide beta-N-acetylglucosaminyltransferase	*Borrelia recurrentis*
	K01924	UDP-N-acetylmuramate--L-alanine ligase	*Borrelia recurrentis*
	K03587	PASTA domain-containing protein	*Borrelia recurrentis*
	K00075	UDP-N-acetylmuramate dehydrogenase	*Borrelia recurrentis*

	K00790	UDP-N-acetylglucosamine 1-carboxyvinyltransferase	*Orientia tsutsugamushi* str. Boryong
	K01000	phospho-N-acetylmuramoyl-pentapeptide-transferase	*Orientia tsutsugamushi* str. Boryong
	K00075	UDP-N-acetylmuramate dehydrogenase	*Orientia tsutsugamushi* str. Boryong
	K00790	UDP-N-acetylglucosamine 1-carboxyvinyltransferase	*Borreliella mayonii*
	K01000	phospho-N-acetylmuramoyl-pentapeptide-transferase	*Borreliella mayonii*
	K06153	undecaprenyl-diphosphate phosphatase	*Borreliella mayonii*
	K01925	UDP-N-acetylmuramoyl-L-alanine--D-glutamate ligase	*Borreliella mayonii*
	K01928	UDP-N-acetylmuramoyl-L-alanyl-D-glutamate--2,6-diaminopimelate ligase	*Borreliella mayonii*
	K00075	UDP-N-acetylmuramate dehydrogenase	*Borreliella mayonii*
	K02563	undecaprenyldiphospho-muramoylpentapeptide beta-N-acetylglucosaminyltransferase	*Borreliella mayonii*
	K03587	transpeptidase family protein	*Borreliella mayonii*
Lysine biosynthesis-	K01928	UDP-N-acetylmuramoyl-L-alanyl-D-glutamate--2,6-diaminopimelate ligase	*Borrelia recurrentis*

	K01929	UDP-N-acetylmuramoyl-tripeptide--D-alanyl-D-alanine ligase	*Borrelia recurrentis*
	K00215	dihydrodipicolinate reductase	*Rickettsia prowazekii* Madrid E
	K01928	UDP-N-acetylmuramoylalanyl-D-glutamate--2,6-diaminopimelate ligase	*Rickettsia prowazekii* Madrid E
	K00928	aspartate kinase	*Orientia tsutsugamushi* str. Boryong
	K00215	4-hydroxy-tetrahydrodipicolinate reductase	*Orientia tsutsugamushi* str. Boryong
	K01928	UDP-N-acetylmuramoyl-L-alanyl-D-glutamate--2,6-diaminopimelate ligase	*Borreliella mayonii*
Quorum sensing-	K15582	MULTISPECIES: ABC transporter permease	*Borrelia recurrentis*
	K15580	peptide ABC transporter substrate-binding protein	*Borrelia recurrentis*
	K03076	preprotein translocase subunit SecY	*Rickettsia prowazekii* Madrid E
	K03217	membrane protein insertase YidC	*Borrelia recurrentis*
	K03076	preprotein translocase subunit SecY	*Orientia tsutsugamushi* str. Boryong

	K03075	preprotein translocase subunit SecG	*Borreliella mayonii*
	K03076	preprotein translocase subunit SecY	*Borreliella mayonii*
	K15580	peptide ABC transporter substrate-binding protein	*Borreliella mayonii*
	K15582	ABC transporter permease	*Borreliella mayonii*
	K03076	preprotein translocase subunit SecY	*Borrelia miyamotoi*
	K15580	peptide ABC transporter substrate-binding protein	*Borrelia miyamotoi*
	K15582	ABC transporter permease	*Borrelia miyamotoi*
Insect hormone biosynthesis pathway	K10718	conserved hypothetical protein	*Ixodes scapularis* (black-legged tick)
	K14938	conserved hypothetical protein	*Pediculus humanus* var. *corporis* (human body louse)
D-Alanine metabolism	K01775	alanine racemase	*Borrelia recurrentis*
	K01775	alanine racemase	*Rickettsia prowazekii* Madrid E
	K01921	D-alanine--D-alanine ligase	*Rickettsia prowazekii* Madrid E

Bacterial chemotaxis	K03412	chemotaxis protein CheB	*Borrelia recurrentis*
	K03413	response regulator	*Borrelia recurrentis*
	K03413	UDP-N-acetylglucosamine 1-carboxyvinyltransferase	*Borreliella mayonii*
	K03406	methyl-accepting chemotaxis protein	*Borreliella mayonii*
	K03413	response regulator	*Borrelia miyamotoi*
Methane metabolism	K01624	class II fructose-bisphosphate aldolase	*Borrelia recurrentis*
	K01624	class II fructose-bisphosphate aldolase	*Borreliella mayonii*
	K01624	class II fructose-bisphosphate aldolase	*Borrelia miyamotoi*
Bacterial secretion system	K03074	protein translocase subunit SecF	*Borrelia recurrentis*
	K03217	membrane protein insertase YidC	*Borrelia recurrentis*
	K03074	preprotein translocase subunit SecF	*Rickettsia prowazekii* Madrid E
	K03076	preprotein translocase subunit SecY	*Rickettsia prowazekii* Madrid E
	K03076	preprotein translocase subunit SecY	*Orientia tsutsugamushi* str. Boryong

	K03118	twin-arginine translocase subunit TatC	*Orientia tsutsugamushi* str. Boryong
	K03199	VirB4 family type IV secretion/conjugal transfer ATPase	*Orientia tsutsugamushi* str. Boryong
	K03201	type IV secretion system protein	*Orientia tsutsugamushi* str. Boryong
	K03201	type IV secretion system protein	*Orientia tsutsugamushi* str. Boryong
	K03075	preprotein translocase subunit SecG	*Borreliella mayonii*
	K03076	preprotein translocase subunit SecY	*Borreliella mayonii*
	K03074	protein translocase subunit SecF	*Borreliella mayonii*
	K03076	preprotein translocase subunit SecY	*Borrelia miyamotoi*
Monobactam biosynthesis	K00215	dihydrodipicolinate reductase	*Rickettsia prowazekii* Madrid E
	K00928	aspartate kinase	*Orientia tsutsugamushi* str. Boryong
	K00215	4-hydroxy-tetrahydrodipicolinate reductase	*Orientia tsutsugamushi* str. Boryong

Lipopolysaccharide biosynthesis	K02517	lipid A biosynthesis lauroyl acyltransferase	*Rickettsia prowazekii* Madrid E
	K00677	UDP-N-acetylglucosamine acyltransferase	*Rickettsia prowazekii* Madrid E
	K02527	3-deoxy-D-manno-octulosonic-acid transferase	*Rickettsia prowazekii* Madrid E
beta-Lactam resistance	K03587	penicillin binding protein (pbpA2)	*Rickettsia prowazekii* Madrid E
	K15580	peptide ABC transporter substrate-binding protein	*Borrelia recurrentis*
	K05366	PBP1A family penicillin-binding protein	*Borrelia recurrentis*
	K03587	transpeptidase family protein	*Borrelia recurrentis*
	K15582	MULTISPECIES: ABC transporter permease	*Borrelia recurrentis*
	K15580	peptide ABC transporter substrate-binding protein	*Borreliella mayonii*
	K15582	ABC transporter permease	*Borreliella mayonii*
	K03587	transpeptidase family protein	*Borreliella mayonii*
	K03587	transpeptidase family protein	*Borrelia miyamotoi*
	K15582	ABC transporter permease	*Borrelia miyamotoi*

Vancomycin resistance	K01775	alanine racemase	*Rickettsia prowazekii* Madrid E
	K01921	D-alanine--D-alanine ligase	*Rickettsia prowazekii* Madrid E
	K01000	phospho-N-acetylmuramoyl-pentapeptide-transferas	*Rickettsia prowazekii* Madrid E
	K02563	undecaprenyldiphospho-muramoylpentapeptide beta-N-acetylglucosaminyltransferase	*Rickettsia prowazekii* Madrid E
	K01775	alanine racemase	*Borrelia recurrentis*
	K01000	phospho-N-acetylmuramoyl-pentapeptide-transferase	*Borrelia recurrentis*
	K01929	UDP-N-acetylmuramoyl-tripeptide--D-alanyl-D-alanine ligase	*Borrelia recurrentis*
	K02563	undecaprenyldiphospho-muramoylpentapeptide beta-N-acetylglucosaminyltransferase	*Borrelia recurrentis*
	K01000	phospho-N-acetylmuramoyl-pentapeptide-transferase	*Orientia tsutsugamushi* str. Boryong
	K01000	phospho-N-acetylmuramoyl-pentapeptide-transferase	*Borreliella mayonii*
	K02563	undecaprenyldiphospho-muramoylpentapeptide beta-N-acetylglucosaminyltransferase	*Borreliella mayonii*

	K01921	D-alanine--D-alanine ligase	*Borrelia miyamotoi*
	K01000	phospho-N-acetylmuramoyl-pentapeptide-transferase	*Borrelia miyamotoi*
	K01775	50S ribosomal protein L11	*Borrelia miyamotoi*
	K02563	undecaprenyldiphospho-muramoylpentapeptide beta-N-acetylglucosaminyltransferase	*Borrelia miyamotoi*
Cationic antimicrobial peptide (CAMP) resistance	K00677	UDP-N-acetylglucosamine acyltransferase	*Rickettsia prowazekii* Madrid E
	K01448	LysM peptidoglycan-binding domain-containing protein	*Borrelia recurrentis*
Ribosome	K02994	transpeptidase family protein	*Borrelia miyamotoi*
	K02933	50S ribosomal protein L6	*Borrelia miyamotoi*
	K02867	50S ribosomal protein L11	*Borrelia miyamotoi*
	K02931	50S ribosomal protein L5	*Borrelia miyamotoi*
	K02895	50S ribosomal protein L24	*Borrelia miyamotoi*
	K02888	50S ribosomal protein L21	*Borrelia miyamotoi*
	K02982	30S ribosomal protein S3	*Borrelia miyamotoi*
	K02881	50S ribosomal protein L18	*Borrelia miyamotoi*

	K02907	50S ribosomal protein L30	*Borrelia miyamotoi*
	K02884	50S ribosomal protein L19	*Borrelia miyamotoi*
	K02864	50S ribosomal protein L10	*Borrelia miyamotoi*
	K02890	50S ribosomal protein L22	*Borrelia miyamotoi*
	K02990	30S ribosomal protein S6	*Borrelia miyamotoi*
Phosphotransferase system (PTS)	K00882	1-phosphofructokinase	*Borrelia recurrentis*
	K02804	PTS transporter subunit EIIC	*Borrelia recurrentis*
Carbon fixation in photosynthetic organisms	K01624	class II fructose-bisphosphate aldolase	*Borreliella mayonii*
D-Amino acid metabolism	K01925	UDP-N-acetylmuramoyl-L-alanine--D-glutamate ligase	*Borreliella mayonii*
Peroxisome	K00869	mevalonate kinase	*Borreliella mayonii*
Cell cycle - Caulobacter	K02313	chromosomal replication initiator protein DnaA	*Borreliella mayonii*
	K02314	replicative DNA helicase	*Borreliella mayonii*
	K03590	cell division protein FtsA	*Borreliella mayonii*
	K02563	undecaprenyldiphospho-muramoylpentapeptide beta-N-acetylglucosaminyltransferase	*Borreliella mayonii*

	K03531	cell division protein FtsZ	*Borreliella mayonii*
Flagellar assembly	K03086	RNA polymerase sigma factor RpoD	*Borreliella mayonii*
	K02419	flagellar type III secretion system pore protein FliP	*Borrelia miyamotoi*
	K03086	RNA polymerase sigma factor RpoD	*Borrelia miyamotoi*
PPAR signaling pathway	K01641	hydroxymethylglutaryl-CoA synthase	*Borrelia miyamotoi*
